# Morphofunctional changes at the active zone during synaptic vesicle exocytosis

**DOI:** 10.15252/embr.202255719

**Published:** 2023-03-06

**Authors:** Julika Radecke, Raphaela Seeger, Anna Kádková, Ulrike Laugks, Amin Khosrozadeh, Kenneth N Goldie, Vladan Lučić, Jakob B Sørensen, Benoît Zuber

**Affiliations:** ^1^ Institute of Anatomy University of Bern Bern Switzerland; ^2^ Department of Neuroscience, University of Copenhagen Copenhagen Denmark; ^3^ Diamond Light Source Ltd Didcot UK; ^4^ Graduate School for Cellular and Biomedical Sciences University of Bern Bern Switzerland; ^5^ Max‐Planck‐Institute of Biochemistry Martinsried Germany; ^6^ BioEM Lab, Biozentrum University of Basel Basel Switzerland

**Keywords:** cryo‐electron tomography, SNARE, synapse, synaptic vesicles, Membranes & Trafficking, Neuroscience

## Abstract

Synaptic vesicle (SV) fusion with the plasma membrane (PM) proceeds through intermediate steps that remain poorly resolved. The effect of persistent high or low exocytosis activity on intermediate steps remains unknown. Using spray‐mixing plunge‐freezing cryo‐electron tomography we observe events following synaptic stimulation at nanometer resolution in near‐native samples. Our data suggest that during the stage that immediately follows stimulation, termed early fusion, PM and SV membrane curvature changes to establish a point contact. The next stage—late fusion—shows fusion pore opening and SV collapse. During early fusion, proximal tethered SVs form additional tethers with the PM and increase the inter‐SV connector number. In the late‐fusion stage, PM‐proximal SVs lose their interconnections, allowing them to move toward the PM. Two SNAP‐25 mutations, one arresting and one disinhibiting spontaneous release, cause connector loss. The disinhibiting mutation causes loss of membrane‐proximal multiple‐tethered SVs. Overall, tether formation and connector dissolution are triggered by stimulation and respond to spontaneous fusion rate manipulation. These morphological observations likely correspond to SV transition from one functional pool to another.

## Graphical abstract

Upon exocytosis triggering, synaptic vesicles at the active zone gain transient tethers, possibly corresponding to super‐priming. In a second phase, the number of inter‐vesicular connections decreases, which may increase vesicle diffusion coefficient and accelerate readily‐releasable pool replenishing.

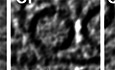

## Introduction

For normal brain functions such as movement coordination or memory formation, communication between neurons is essential. In the central nervous system, neurons communicate through the release of neurotransmitters at synapses. This process relies on synaptic vesicle (SV) exocytosis, that is, the fusion of neurotransmitter‐filled SVs with the plasma membrane (PM). SV exocytosis involves a sequence of steps (Verhage & Sørensen, 2008; Südhof Thomas, 2013). Namely the vesicle is brought to the active zone (AZ) PM and the exocytosis machinery goes through a maturation process, termed priming, which renders the vesicle able to undergo exocytosis immediately upon stimulation. These SVs form the readily releasable pool (RRP). Finally, a calcium influx triggers fusion of the SV with the PM.

In electron microscopy (EM) of resin‐embedded, heavy‐metal‐stained samples, Docked SVs are defined as SVs in very close proximity or direct contact with the PM, whereas priming refers to SV ability to undergo exocytosis immediately upon stimulation. Whether every docked SV is also primed has been debated (Verhage & Sørensen, 2008; Imig *et al*, 2014; Kaeser & Regehr, 2017). A high‐pressure freezing/freeze‐substitution EM study of synapses has indicated that vesicles that are in direct contact with the PM, that is, docked, are also primed and belong to the RRP and that this situation occurs downstream of vesicle tethering (Imig *et al*, 2014).

From a molecular perspective, priming involves several proteins, including the SNARE complex (SNAP‐25, syntaxin‐1, and synaptobrevin‐2), Munc13, Munc18, synaptotagmin‐1, and complexin (Südhof Thomas, 2013; Rizo & Xu, 2015). All three SNAREs form a highly stable, tight four‐helix bundle, known as trans‐SNARE complex. The surfaces of the SV and the PM are both negatively charged and therefore tend to repulse each other. The formation of the trans‐SNARE complex counteracts this repulsion and brings the SV and the PM in high proximity (Sutton *et al*, 1998). Evidence has suggested that the SNARE complex is only partially zipped in primed vesicles (Sørensen *et al*, 2006). Furthermore, various studies have suggested that the formation of at least three SNARE complexes provides the necessary energy for a vesicle to become fusion competent (Domanska *et al*, 2009; Mohrmann *et al*, 2010; Shi *et al*, 2012). Yet in the absence of cytoplasmic Ca^2+^, minimal spontaneous exocytosis takes place. When the pre‐synaptic terminal gets depolarized by an action potential, Ca^2+^ flows in the cytoplasm and binds to synaptotagmin‐1, which is localized at the SV surface. Upon Ca^2+^ binding, synaptotagmin‐1 was proposed to insert between the head groups of the PM anionic phospholipids and trigger membrane curvature and destabilization, leading first to hemifusion and subsequently to fusion (McMahon *et al*, 2010). Interestingly, much of the trans‐SNARE bundle surface is negatively charged. This contributes to the electrostatic barrier that minimizes spontaneous fusion. Synaptotagmin‐1 can then act as an electrostatic switch that triggers exocytosis. Introducing negatively charged side chains to SNAP‐25 by site‐directed mutagenesis reduces the rate of spontaneous and evoked exocytosis, whereas introducing more positive side chains enhances the rate of spontaneous exocytosis and depletes the RRP (Ruiter *et al*, 2019).

Cryo‐electron tomography (cryo‐ET) allows imaging of fully hydrated, vitrified samples where structural information is preserved to atomic resolution. It revealed that under resting conditions, SVs are rarely in direct contact with the PM and the majority of AZ‐proximal SVs are connected to the PM by a variable number of short tethers (Fernández‐Busnadiego *et al*, 2010; Zuber & Lučić, 2019). The observed gap between the SV and the PM is consistent with the model of an electrostatic barrier formed by the negative charges of the SV, the PM, and the trans‐SNARE bundle (Ruiter *et al*, 2019). In synaptosomes treated with hypertonic sucrose solution, which is known to deplete the RRP, the majority of tethered vesicles had only 1 or 2 tethers (Rosenmund & Stevens, 1996; Ashton & Ushkaryov, 2005; Fernández‐Busnadiego *et al*, 2010). This observation suggested that the RRP consists of SV, which is linked to the PM by three or more tethers. The RRP, as identified by morphological criteria, only represents a minority of AZ‐proximal vesicles. This is in agreement with previous reports. In one of them, the term pre‐primed pool was used for the few vesicles (~1 vesicle at hippocampal synapses) that are rapidly released. Another publication showed that the RRP is made up of only 10–20% of SVs located on the AZ (equal to ~1 vesicle on hippocampal synapses) (Hanse & Gustafsson, 2001; Moulder, 2005). The ensemble of proximal vesicles that are not in the RRP has been termed non‐RRP and presumably belong to the recycling pool that releases more slowly (Denker & Rizzoli, 2010; Fernández‐Busnadiego *et al*, 2010).

Farther away from the AZ, partially inter‐mixed with the recycling pool, is the reserve pool containing vesicles that only release upon high‐frequency stimulation. Vesicles in the reserve pool are tightly clustered and well interconnected by structures that were termed connectors (Denker & Rizzoli, 2010; Fernández‐Busnadiego *et al*, 2010). It should be noted that the molecular nature of connectors is not known and is possibly heterogeneous. Synapsin has been proposed as a molecular constituent. However, since the deletion of all forms of synapsin does not lead to the complete absence of connectors, it is clear that not all connectors contain synapsin (Hirokawa *et al*, 1989; Siksou *et al*, 2007). The second row of SVs near the active zone (45–75 nm from AZ), immediately after the proximal vesicles (< 45 nm from AZ), is called the intermediate region. Resting‐state intermediate SVs are less densely packed and also less connected than proximal SVs (Zuber & Lučić, 2019). This suggests that, after exocytosis of RRP SVs, intermediate SVs could be rapidly recruited in the RRP by diffusion (Rothman *et al*, 2016). Synaptic activity enhances the mobility of a fraction of SVs, whereas it induces synapsin dissociation from SVs in a synapsin phosphorylation‐dependent manner (Chi *et al*, 2001; Forte *et al*, 2017). The same mobility enhancement can be achieved through inhibition of synapsin dephosphorylation, which leads to synapsin dissociation from SVs, or by knocking out all three synapsin forms (Benfenati *et al*, 1989; Jordan *et al*, 2005; Orenbuch *et al*, 2012). Interestingly, ribbon synapses do not express synapsin and show higher SV mobility than conventional synapses (Holt *et al*, 2004). It is, therefore, conceivable that inter‐SV connectors restrain SV diffusion and that synaptic activity influences the level of inter‐SV connectivity and thereby their mobility.

To investigate this hypothesis and to better understand the impact of depolarization and synaptic activity on SV tethering, we designed two sets of cryo‐ET experiments. On the one hand, we compared the morphology of wild‐type rat synaptosomes in resting state and a few milliseconds after depolarization. On the other hand, to study the consequences of increased or decreased spontaneous synaptic activity, we imaged synapses in mouse neuronal culture expressing either wild‐type SNAP‐25, a more positively charged SNAP‐25 mutant (4K mutant), or a more negatively charged, constitutively active mutant of SNAP‐25 (4E mutant) (Ruiter *et al*, 2019). The more positively charged SNAP‐25 mutant showed no triple‐tethered SV, confirming the morphological definition of the RRP (Ruiter *et al*, 2019). Our experiments revealed an immediate formation of additional tethers between proximal RRP vesicles and the PM after depolarization. Shortly after exocytosis, the level of inter‐SV connectivity was decreased among SVs situated in a 25–75 nm distance range from the AZ PM. Altogether, our results indicate a regulation through connectors of SV mobility and their recruitment at the AZ PM.

## Results

To analyze the morphological changes occurring in the pre‐synapse shortly after stimulation, we pursued a time‐resolved cryo‐electron tomography approach similar to the one introduced by Heuser *et al* (1979). Whereas Heuser *et al* (1979) used an electrical stimulus, we chose to trigger exocytosis by spraying a depolarizing solution containing 52‐mM KCl onto the specimen a few milliseconds before freezing for two reasons. First, spraying allows cryo‐ET imaging because it is compatible with plunge freezing directly on an EM grid, which is not the case with electrical stimulation. Second, this method allows catching synapses at delays between stimulation and freezing lower than a millisecond, as explained below. Some of the delays achieved here are shorter than those attained by electrical stimulation. The solution was sprayed with an atomizer and droplets hit the EM grid a few milliseconds before freezing. The spray‐mixing plunge‐freezing setup was custom built based on a system introduced by Berriman and Unwin (1994).

**Figure 1:**
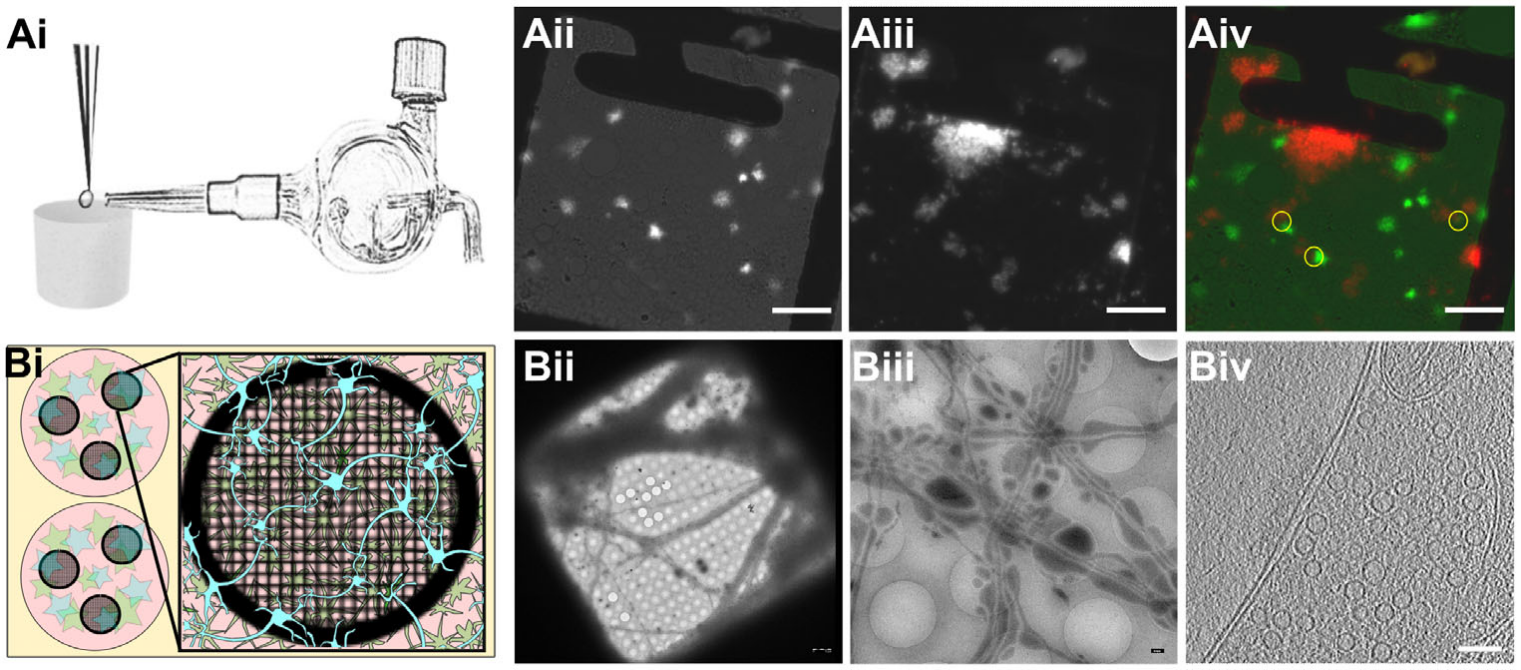
Experimental models Ai
Glass atomizer used to disperse depolarizing solution on the EM grid milliseconds before the grid is vitrified.Aii
Spray droplets imaged with the GFP filter set. Scale bars, 20 μm.Aiii
Synaptosomes imaged with the DAPI filter set. Scale bar, 20 μm.Aiv
Overlay of spray droplets (green) and synaptosomes (red). Yellow circles show contact between droplets and synaptosomes in thin area, which is suitable for cryo‐ET. Scale bar, 20 μm.Bi
Schematic drawing of a six‐well Petri dish depicting astrocytes (pink) growing at the bottom of the Petri dish below EM grids (black, round grid overlaying the astrocytes), with neurons (blue) growing on top of the grids.Bii
Grid square overview with neurons growing over it. Scale bar, 5 μm.Biii
Medium‐magnification overview of neurons growing over R2/1 holes. Scale bar, 500 nm.Biv
One slice of a tomogram depicting a synapse. Scale bar, 100 nm.

**Figure 2:**
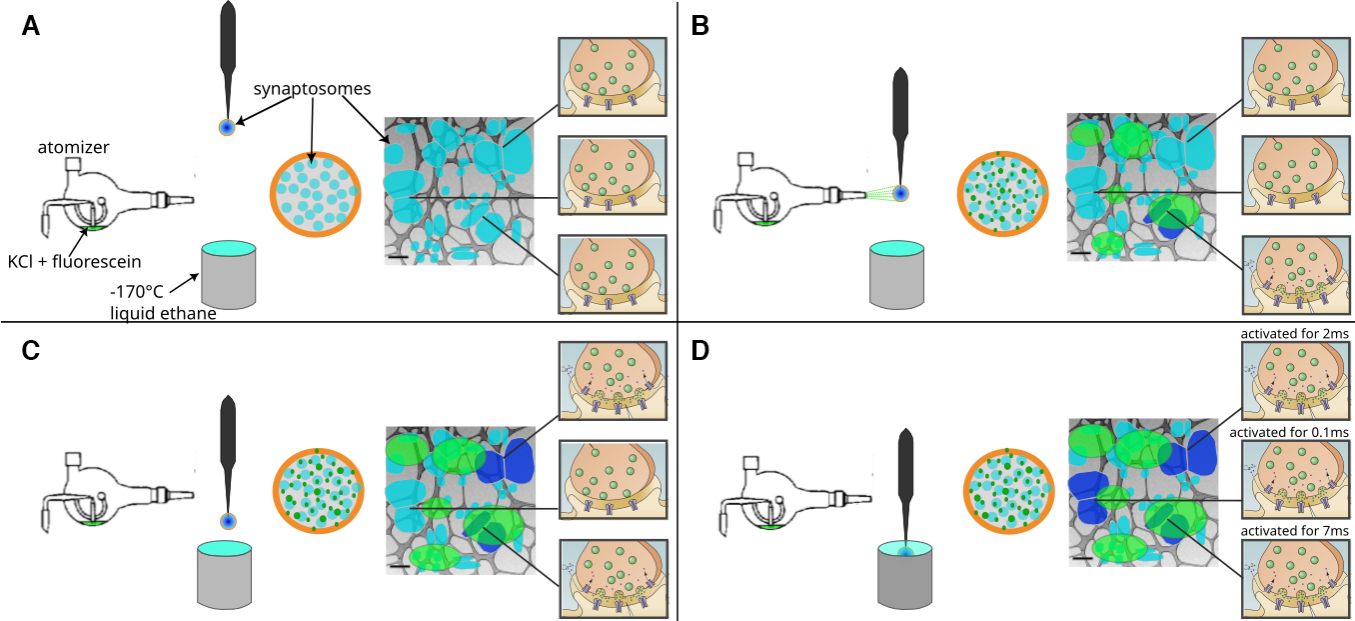
Schematic representation of a spray‐mixing plunge‐freezing experiment A–D
In a single experiment, different synaptosomes get stimulated for between < 1 and 7 ms.** An EM grid is held by tweezers and is covered with synaptosomes in HBM solution. A magnified view of a grid square shows synaptosomes in blue and their synaptic state of three synaptosomes is represented on the rightmost part of each panel. Panel (A) represents the situation immediately after blotting off the solution excess before the grid is sprayed. The grid is accelerated towards the cryogen. Panel (B) shows a snapshot of the experiment when the grid crosses the spray, 7 ms before the freezing. Some fluorescently dyed droplets containing HBM with 52 mM KCl land on the grid and are depicted in green. At this time point, a synaptosome located at the impact point of a droplet is activated and is depicted in dark blue. Panel (C) shows a snapshot 5 ms later, that is, 2 ms before freezing. As KCl diffuses away from droplet impact points, another synaptosome gets activated because locally KCl concentration has reached a concentration to depolarize the synaptosome sufficiently so that voltage‐gated calcium channels open. Panel (D) shows a synaptosome at the time of immersion with ethane. 0.1 ms before freezing a third synaptosome got exposed to a high enough concentration of KCl and got stimulated.

**Figure EV1:**
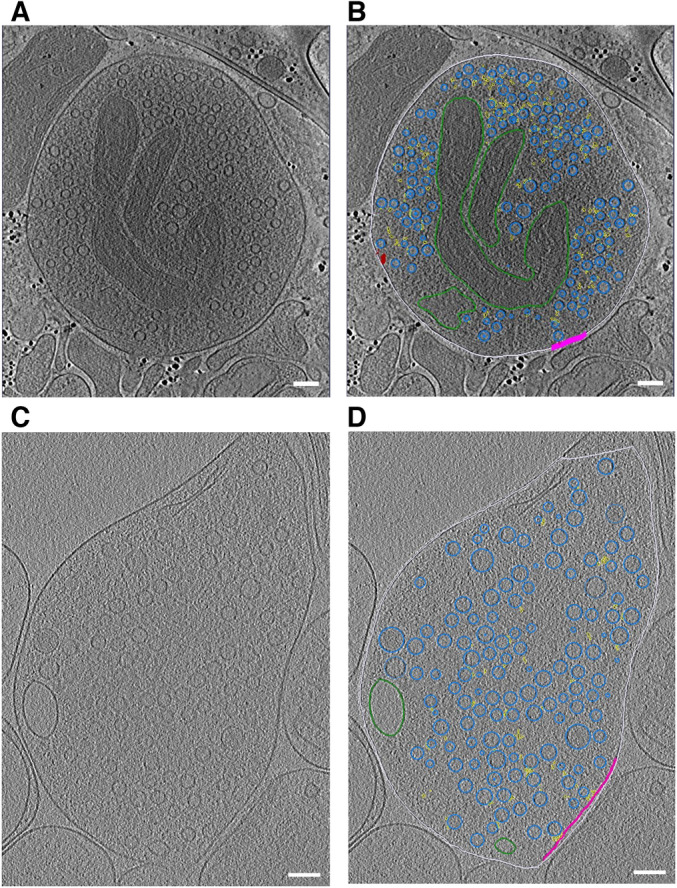
Representative slices through tomograms A, B
Tomographic slice without (A) and with (B) segmentation of synaptosome with late‐fusion events.C, D
Tomographic slice without (C) and with (D) segmentation of WT SNAP‐25 neurons. Data information: segmentation colors: off‐white = cell outline; pink = active zone; blue = synaptic vesicles; green = mitochondria; yellow = connectors and red = tethers. Scale bar, 100 nm.

The spray droplet size was optimized by cutting a 1 ml pipette tip to a diameter matching an EM grid (3 mm) and fixed to the atomizer glass outlet to disperse the spray (Fig 1Ai). Furthermore, to achieve the shortest possible delay between spraying and freezing, the nozzle was set 1–2 mm above the liquid ethane container. This generated many small spray droplets spread throughout the grid (Figs 1Aii–Aiv and 2A–D). Even if sprayed droplets were well distributed throughout the grid, not all synaptosomes were in contact with exocytosis‐triggering KCl solution. Synaptosomes located on the landing spot of a droplet were stimulated instantly, and therefore, were frozen for a delay equal to the time between the grid crossing the spray and hitting the cryogen, which was typically set at 7 or 35 ms (Fig 2B). However, synaptosomes situated at a distance from a landing spot were only stimulated when the KCl concentration rose due to diffusion reaching a threshold triggering voltage‐gated calcium channel opening (Fig 2C and D). Through this process, we were able to trap stimulated synapses at the very earliest stages of exocytosis. Given the very low throughput of cryo‐electron tomography, we followed a correlative light and electron microscopy approach. By cryo‐fluorescence microscopy, we identified areas where synaptosomes fluorescently labeled by calcein blue and spray droplets labeled by fluorescein were colocalized. Additionally, phase contrast imaging enabled quality control of the frozen EM grid with respect to ice contamination and ice cracks, as shown previously (Sartori *et al*, 2007). Nine control and nine stimulated synaptosome tomograms were analyzed (Fig EV1A and B, Movie EV1, Table EV1). We restricted our analysis to synaptosomes that possessed a smooth PM, free of signs of rupturing, and that had a mitochondrion, as we considered these factors essential for synaptosome function.

**Figure EV2:**
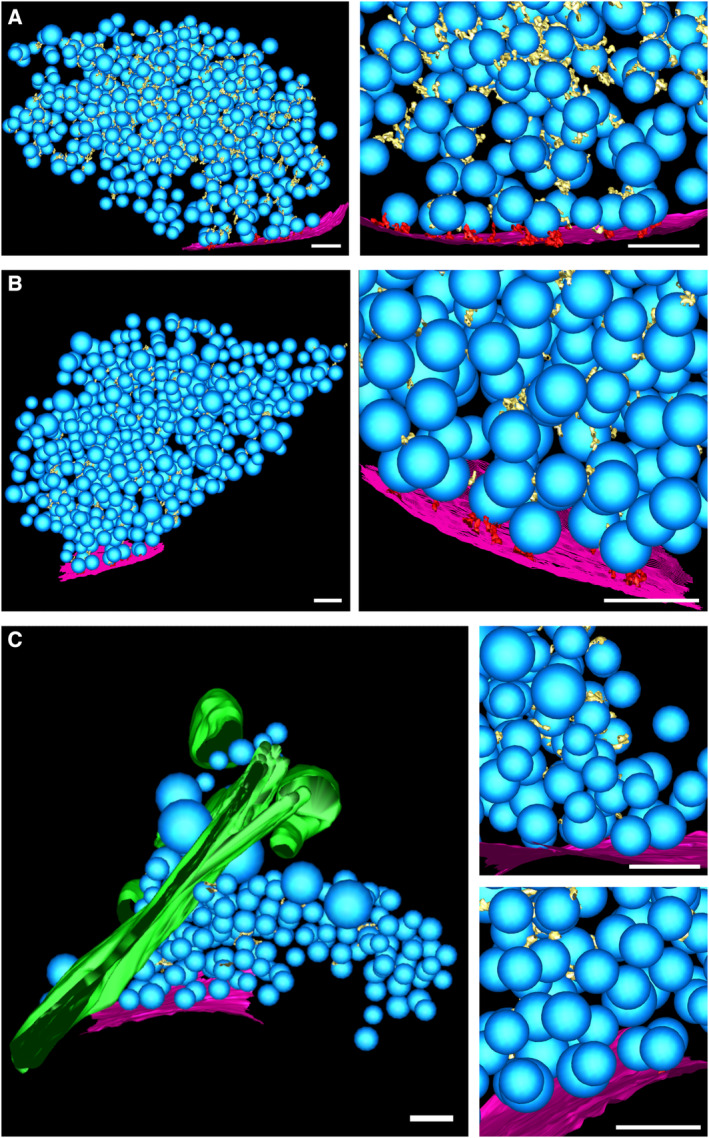
3‐D rendered segmented tomograms of neuron synapses A–C
(A) SNAP‐25 WT, (B) SNAP‐25‐4E, and (C) SNAP‐25‐4K. (left) Overview, (right) detail. Blue: synaptic vesicles; purple: active‐zone plasma membrane; green: endoplasmic reticulum‐like organelle; yellow: connectors; and red: tethers. Scale bars: 100 nm.

In addition, we manipulated the electrostatic state of the SNARE complex through mutated SNAP‐25 protein introduced using lentiviral vectors into primary SNAP‐25 knockout neurons grown on EM grids (Ruiter *et al*, 2019) (Fig 1Bi–Biv). The “4E” mutation contains four glutamic acid substitutions, whereas the “4K” contains four lysine substitutions; all mutations are placed in the second SNARE motif of SNAP‐25 and were shown to decrease and increase the rate of spontaneous miniature release, respectively (Ruiter *et al*, 2019). Optimization of primary neuron culturing conditions allowed us to establish a protocol, which provides functional synapses thin enough for direct imaging by cryo‐ET. Astrocytes were added to 12‐well plates and were grown for 2 days. After 2 days, the medium was exchanged for a medium that favors neuronal growth and impedes astrocyte growth. At the same time, a droplet of the neuronal suspension was added onto a flame‐sterilized EM grid and incubated for 30 min at 37°C, thereafter the grids were placed into the 12‐well plates containing the astrocytes. Neurons were grown for 10–14 days until plunge freezing and were then analyzed at a Titan Krios by cryo‐ET (Figs EV1C and D, and EV2A–C, Movie EV2, Table EV2). Thereby, we could image chronically overactive or inactive synapses and relate pre‐synaptic architectural modifications to different functional states.

### Increased membrane curvature at the onset of exocytosis

We first analyzed the morphology of SVs fusing with the AZ PM. As explained above, synaptosomes of a single grid have not all been stimulated for the same duration (Fig 2). The time interval between triggering exocytosis and freezing ranged between 0 ms and the interval between spray droplets hitting the grid and freezing (maximum 35 ms; see Berriman & Unwin, 1994). This offered the unique possibility to observe SV exocytosis events immediately after their initiation, and even before membranes have started to mix.

Synaptosomes from both control and sprayed grids were thoroughly analyzed for signs of exocytosis, which consisted of morphological changes in the AZ PM and the SV membrane occurring upon stimulation. These signs were only detected in synaptosomes from sprayed grids and neither in non‐sprayed tomograms acquired specifically for this study nor in past studies containing hundreds of SVs at the active zone.

**Figure 3:**
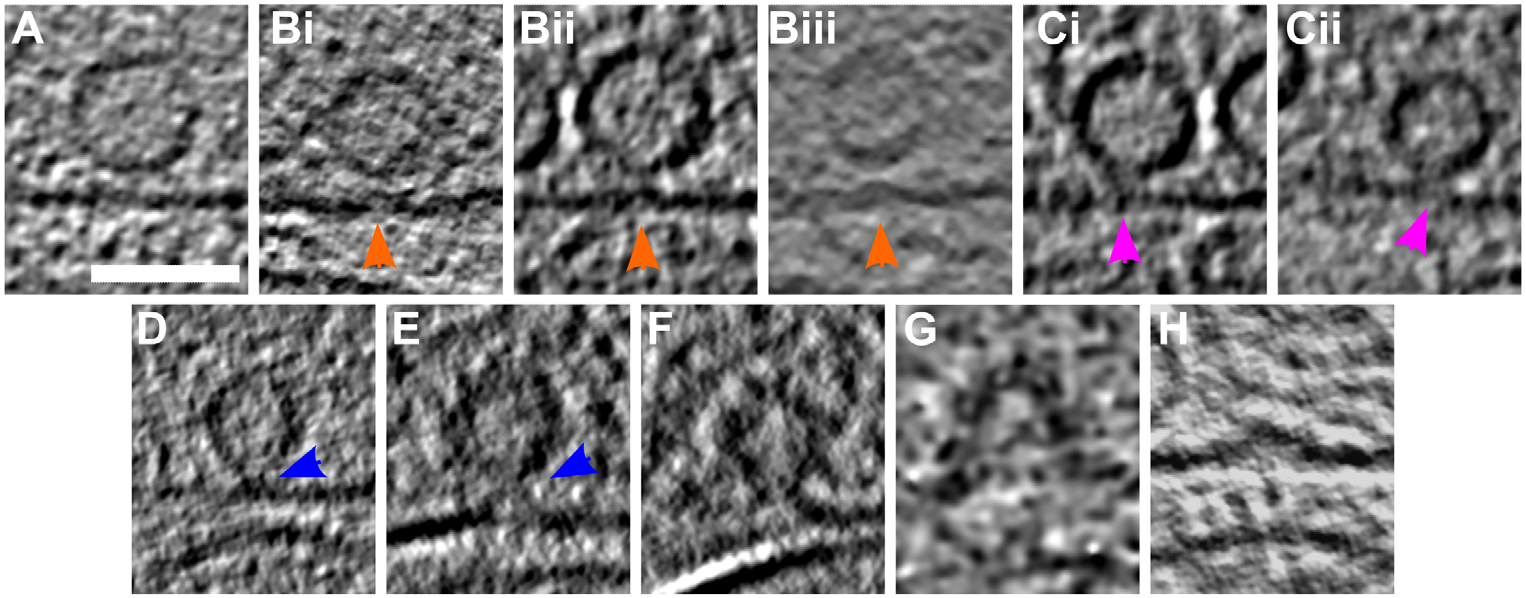
SV exocytosis morphology A–H
Tomographic slice of non‐stimulated (A) and stimulated rat synaptosomes (B–H). (A) Image of a 2.2‐nm‐thick tomographic slice showing a non‐stimulated with SVs at the AZ and a straight PM. (Bi) Membrane curvature event, 2.2‐nm‐thick tomographic slice. (Bii) Membrane curvature event, 6.5‐nm‐thick tomographic slice. (Biii) Membrane curvature event, 2.24 nm‐thick tomographic slice. Orange arrows showing membrane curvature event. (Ci, Cii) Lipid perturbations of PM and SV, 22‐nm‐thick tomographic slices. The space between SV and PM is denser than in the non‐stimulated synaptosomes (see pink arrow). (D–F) Vesicles with a pore opening that might be on the way to full‐collapse fusion, 33‐nm‐thick tomographic slice thickness: 22 nm (D), 30.8 (E), and 33 nm (F). (G) Wide pore opening, most likely on the way to full‐collapse fusion, 2.2 nm tomographic slice. (H) Remaining bump at the end of full‐collapse fusion, 11‐nm‐thick tomographic slice. Scale bar, 50 nm. Total number of observations of each type of exocytosis events: (B) 8; (C) 3; (D) 3; (E) 2; (F) 3; (G) 1; and (H) 11. Events of type (B–E) were classified as early, while events of type (F–H) were classified as late.

In non‐sprayed synaptosomes, proximal SVs were spherical and the opposite plasma membrane was smooth (Fig 3A). Among the synapses that showed bent lipid membranes, we distinguished early‐ and late‐fusion stages. The early‐stage events were defined as those that do not show open fusion pore. We observed two different types of these events, as follows: (i) Both the vesicle membrane and the PM were slightly bent toward each other, without making a contact (Fig 3Bi–Biii; orange arrows; *n* = 8). These structures, which have previously been reported in liposomes but not in synapses, have been referred to as membrane curvature events (McMahon *et al*, 2010). Control synaptosomes (i.e., not sprayed), on the other hand, had a straight PM and no SV membrane was buckled (Fig 3A). (ii) A contact was formed between the vesicle and the PM bilayer where both membranes lose their clear contours (Fig 3Ci and Cii; pink arrows; *n* = 3), or where they establish a point contact (Fig 3D and E; blue arrows; *n* = 5). Similar events were observed previously only by cryo‐ET in chronically stimulated synaptosomes, but could not be firmly associated with a particular SV exocytosis state (Fernández‐Busnadiego *et al*, 2010).

The late‐fusion stage events were defined as those where SV lumenal and the extracellular regions were in direct contact. These events include small SV fusion pore opening (Fig 3F; blue arrows; *n* = 3), wide fusion pore opening (Fig 3G; *n* = 1), and cases where SVs collapsed almost completely and only a small bump on the PM remained visible (Fig 3H; *n* = 11). Using high‐pressure freezing and freeze substitution, Imig *et al* (2014) observed very similar structures in mouse hippocampal organotypic slices. Nevertheless, very fine lipid membrane deformations such as those in Fig 3Bi–Biii and SVs located at a few nanometer distances to the AZ PM were not observed, possibly because of the application of dehydration and heavy metal staining.

The notion that the SV fusion pore opening is the last step before the full SV collapse is well established, and it was hypothesized that lipid membrane perturbation and/or lipid point contact precede pore opening (Rizo, 2018). Therefore, our temporal assignment of the early‐ and late‐fusion stages is dictated by the current understanding of neurotransmitter release. Regarding the temporal assignment of events comprising the early and late stages, it is very likely that lipid membrane perturbation events precede establishing the contact between SV and PM lipids (Fig 3B–E). Also, it is expected that in the full‐collapse fusion, the SV pore progressively widens (Fig 3F–H). These establish the most likely temporal sequence of the events we observed.

Importantly, in the analysis that follows, only the early‐ and late‐fusion stages are distinguished. Furthermore, while the fusion stages were defined based on the observed lipid deformations, further analysis concerning features that were not used to define the fusion stages, namely SV‐bound protein complexes and the position of SVs within the pre‐synaptic terminal.

### Synaptic vesicle distribution is impacted by synaptic activity

**Figure 4:**
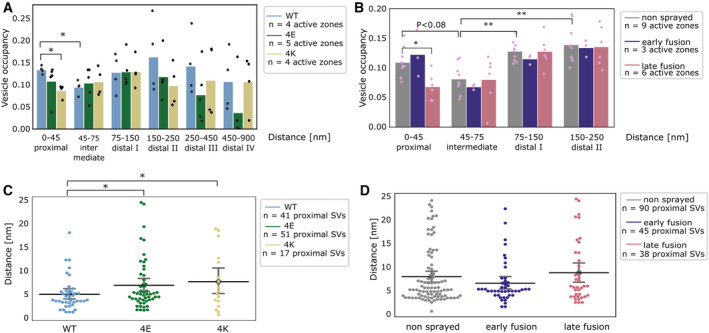
SV distribution A, B
Vesicle occupancy expressed as fraction of cytosol volume occupied by vesicles as a function of distance to AZ in (A) cultured neurons and (B) synaptosomes. Each bar is the average value of a distance group of all tomograms of the same treatment/genotype. Each dot represents the occupancy of a distance group in a single tomogram. Statistical test: multiple all against reference pairwise ANOVA comparisons with Benjamini–Hochberg correction. *P < 0.05 and **P < 0.01 after Benjamini–Hochberg correction. The reference distance group was the intermediate one. The reference experimental conditions were the WT genotype (A) and non‐sprayed synaptosomes (B), respectively. Comparisons between distance groups were performed only within the reference experimental conditions.C, D
Distance of proximal SVs from the AZ. Each dot represents the value of an individual SV. Horizontal line: mean; whiskers: 2×SEM interval. Statistical test: multiple all‐against‐reference pairwise ANOVA comparisons with Benjamini–Hochberg correction; *P < 0.05 after Benjamini–Hochberg correction.

Non‐sprayed rat synaptosomes as well as WT‐SNAP‐25 mouse cultured neuron synapses showed typical SV distribution, as observed in previous cryo‐ET studies (Fig 4) (Fernández‐Busnadiego *et al*, 2010). Vesicle occupancy in WT‐SNAP‐25 synapses was 0.13 in the proximal zone (0–45 nm from the AZ PM), dropped to 0.09 in the intermediate zone (45–75 nm), rose to 0.12 in the distal I zone (75–150 nm), rose further to 0.16 in the distal II zone (150–250 nm), decreased to 0.14 in the distal III zone (250–450 nm), and decreased further to 0.10 in the distal IV zone (450–900 nm) (Fig 4A).

The absolute values differ between WT‐cultured mouse neurons and non‐stimulated rat synaptosomes, but the SV occupancy distribution follows the same pattern. The difference in absolute values can likely be attributed to the different experimental and animal models used. Sprayed synaptosomes that were showing early signs of exocytosis had a nearly identical SV occupancy pattern as non‐sprayed synaptosomes (Fig 4B, dark blue and gray, respectively). However, when SV full‐collapse figures were apparent, SV occupancy in the proximal zone was reduced (*P* < 0.08 with Benjamini–Hochberg correction), whereas SV occupancy further away from the AZ PM was unchanged. This is similar but less pronounced than the reduction of the proximal SV occupancy observed after prolonged stimulation (30–60 s), which we reported earlier (Fernández‐Busnadiego *et al*, 2010). Thus, it is consistent with some membrane‐proximal SVs having engaged in exocytosis, while none of the recycling and reserve pool SVs have.

In order to investigate the consequences of chronic high or low synaptic activity, we investigated the 4E and 4K mutants (Fig 4A, green and gold, respectively). In the proximal zone, SV was significantly less concentrated in the constitutive active 4K mutant than in the WT (*P* < 0.05, ANOVA test with Benjamini–Hochberg correction). This can be readily attributed to the high probability of spontaneous exocytosis generated by the additional positive charges of the SNARE bundle. Furthermore, the proximal SVs of both mutants were located significantly further away from the active‐zone plasma membrane (*P* < 0.05 in both cases, ANOVA test with Benjamini–Hochberg correction, Fig 4C). The larger mean distance between proximal SVs and plasma membrane in the 4E mutant might result from the repulsion between negative charges present in SNAP‐25 and on the plasma membrane. In the case of the 4K mutant, the larger mean distance may be due to the high spontaneous exocytotic activity of this mutant. Indeed, SVs located in close proximity to the plasma membrane have a very high probability of fusing, which then leaves proximal SVs located at a higher distance to the PM. Of note, no significant difference in the mean distance between proximal SVs and the plasma membrane was observed following stimulation in rat synaptosomes (Fig 4D). In the most distal zones of neuronal synapses, SV occupancy in both mutants mostly got lower than in the WT, although the differences were not significant (Fig 4A).

### Proximal vesicles form additional tethers following stimulation

**Figure 5:**
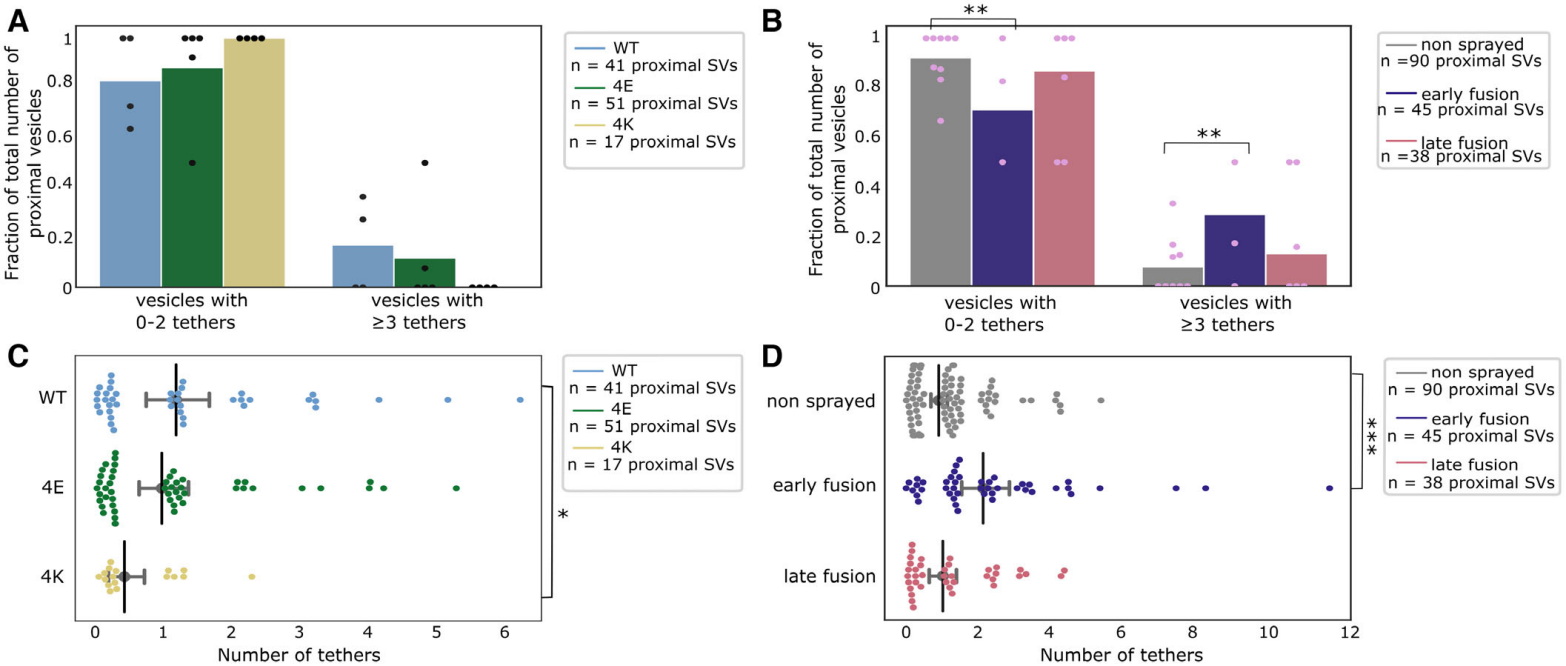
Proximal SV tethering A, B
Fraction of proximal SVs that are triple tethered. Each bar shows the overall fraction of all proximal SVs from a given experimental condition. Each dot represents the value of an individual active zone. Statistical test: multiple all‐against‐control pairwise χ^2^‐test comparisons with Benjamini–Hochberg correction. **P < 0.01 after Benjamini–Hochberg correction. The reference was the WT genotype (A) or non‐sprayed synaptosomes (B).C, D
Number of tethers per proximal SV. Each dot represents an individual SV. The vertical line represents the mean value, and the horizontal whiskers correspond to the 95% confidence interval. Statistical test: multiple all‐against‐control pairwise ANOVA comparisons with Benjamini–Hochberg correction; *P < 0.05 and ***P < 0.001, after Benjamini–Hochberg correction.

**Figure EV3:**
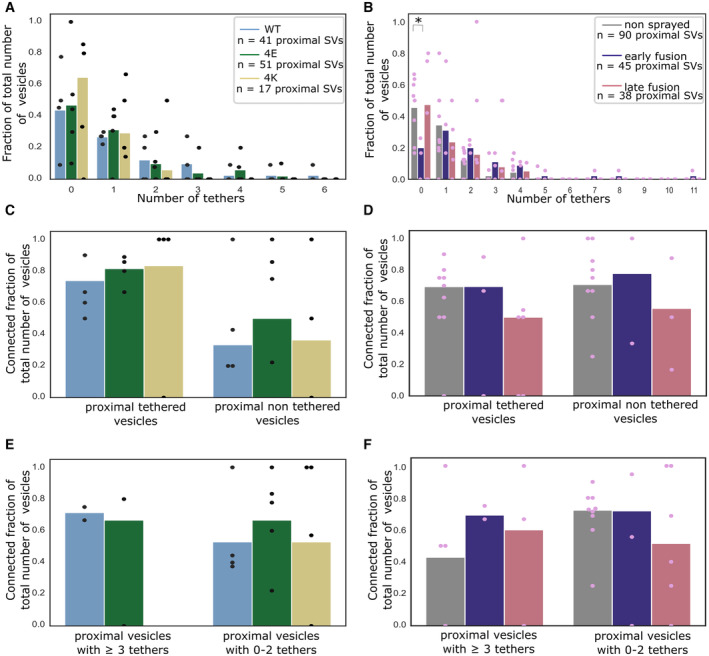
Additional SV tethering and connectivity data A, B
Histogram of the number of tethers per proximal SV. Statistical test: pairwise χ^2^ test between control and each experimental condition in the zero‐tether group with Benjamini–Hochberg correction. *P < 0.05.C, D
Histogram of connected SV among tethered or non‐tethered proximal SVs.E, F
Histogram of connected SV among proximal non‐RRP or RRP SVs. Data information: (A, C, E) Synapses in mouse cultured neurons. (B, D, F) Rat synaptosomes.

We investigated the tethering state of proximal SVs (i.e., the SVs whose center is located within 45 nm of the AZ PM; Fig 5A–D). We first compared the situation in synaptosomes prior to and following stimulation. In non‐sprayed synaptosomes, 54% of the proximal vesicles were tethered, which is in agreement with previous results (Fig EV3B) (Fernández‐Busnadiego *et al*, 2010). Interestingly, in the early fusion group, the fraction of tethered proximal vesicles significantly increased to 80% (*P* < 0.05, χ^2^ test with Benjamini–Hochberg correction). In the late‐fusion group, however, 53% of the proximal vesicles were tethered, which was not significantly different from the non‐sprayed group. The average number of tethers per proximal SV followed the same pattern. Proximal SVs had 0.89 ± 0.12 tethers in the non‐sprayed group (Fig 5D). This parameter significantly rose to 2.09 ± 0.33 in the early fusion group (*P* < 0.001, ANOVA test with Benjamini–Hochberg correction), while it returned to 1.00 ± 0.20 in the late‐fusion group.

We then analyzed whether the decreased occupancy in the late‐fusion group was associated with a decreased number of triple‐tethered SVs (defined as SV with at least three tethers), which as mentioned in the introduction are suggested to belong to the RRP. In resting, non‐sprayed synapses, 8% of the proximal SVs were triple tethered (Fig 5B). Surprisingly, the fraction of triple‐tethered proximal SVs drastically increased to 29% in the early fusion group (*P* < 0.01, χ^2^ test with Benjamini–Hochberg correction). The fraction decreased to 13% in the late‐fusion group. This suggests that upon stimulation, some proximal SVs very rapidly acquire new tethers. Using our definition of the RRP (vesicles that are triple tethered), this would indicate that the RRP rapidly increases after stimulation and more vesicles become primed for exocytosis. Furthermore, the lower proximal vesicle occupancy in the late‐fusion group indicates that under our stimulation conditions, replenishing vesicles to the proximal zone is slower than their release.

The situation in the WT‐SNAP‐25 neurons was similar to unstimulated synaptosomes. Fifty‐three percent of the proximal SVs were tethered and 17% of the proximal SVs belonged were triple tethered (Figs EV3A and 5A). On average, proximal SVs had 1.17 ± 0.23 tethers (Fig 5C). The corresponding values for the 4E mutants were not significantly different (15% and 0.96 ± 0.18, respectively). However, in all 4K mutant datasets, there was not a single SV that was part of the RRP, that is, triple tethered. Similarly, the number of tethers per proximal SV was significantly lower in the 4K mutant than in the WT (Fig 5C, *P* < 0.05, ANOVA test with Benjamini–Hochberg correction). These results are in line with physiological measurements that have shown that the RRP is depleted in the chronically spontaneously active 4K mutant, and they provide additional evidence that RRP vesicles have at least three tethers (Ruiter *et al*, 2019).

### Synaptic activity modifies inter‐SV connectivity

**Figure 6:**
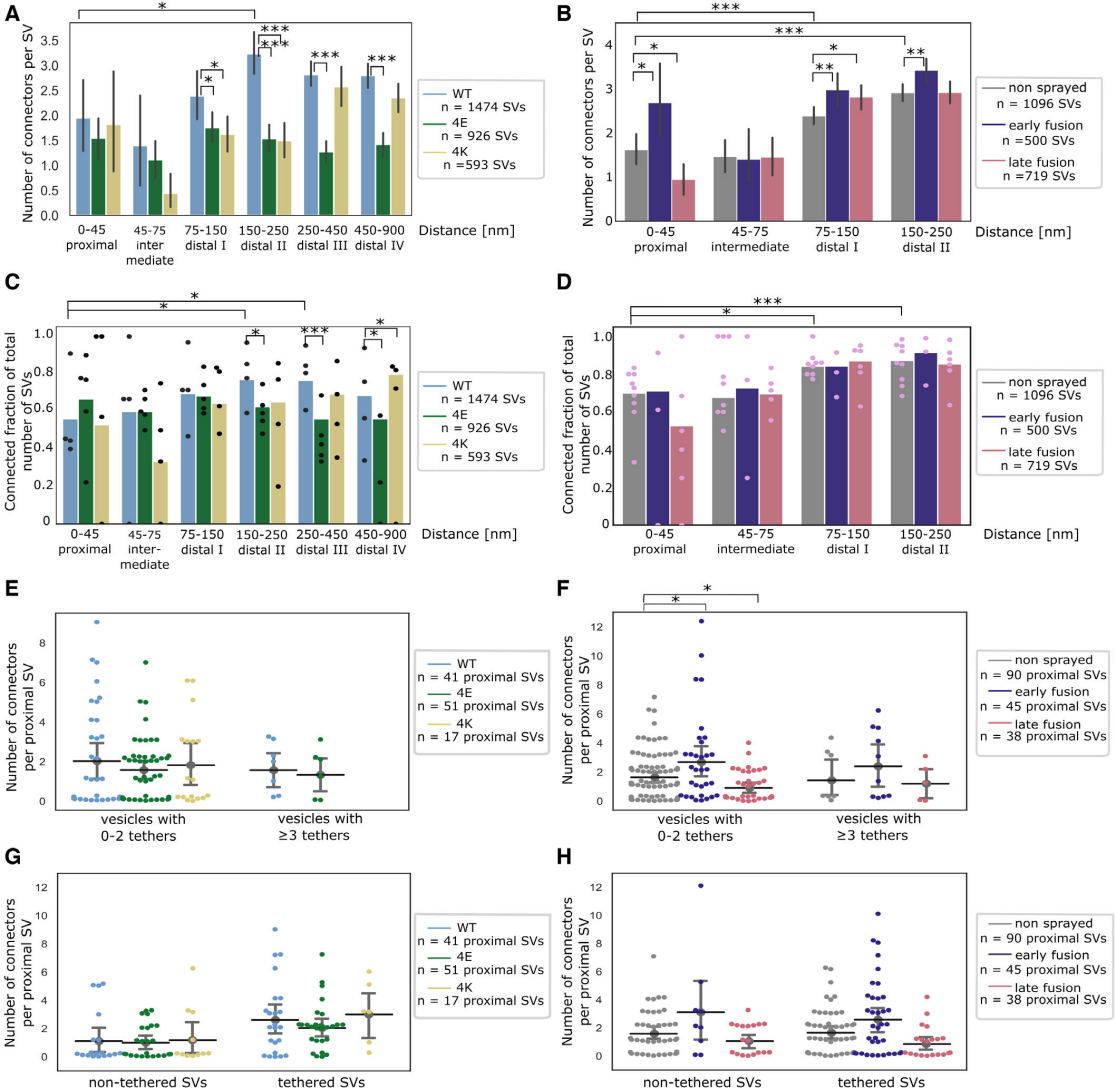
SV connectivity A, B
Number of connectors per SV as a function of their distance to the AZ PM for mouse neurons (A) and rat synaptosomes (B). Each bar represents the mean value of all SVs of the subgroup. The lines represent 2×SEM intervals. Statistical tests: multiple all‐against‐reference pairwise ANOVA comparisons with Benjamini–Hochberg correction. Within a single experimental condition, the reference was the proximal distance group; within a single distance group, the reference was the WT genotype (A) or no‐sprayed synaptosomes (B).C, D
Fraction of connected vesicles as a function of distance to the AZ PM for mouse neurons (C) and rat synaptosomes (D). Each bar shows the overall fraction of all SVs in a given distance group and a given experimental condition. Each dot represents the corresponding value of an individual active zone. Statistical test: multiple all‐against‐reference pairwise χ^2^ test with Benjamini–Hochberg correction; references were defined as in (A) and (B).E, F
Number of connectors per proximal SV not belonging or belonging to the RRP for mouse neurons (E) and rat synaptosomes (F). Horizontal line: mean; whiskers: 2×SEM interval.G, H
Number of connectors per non‐tethered or tethered proximal SV for mouse neurons (G) and rat synaptosomes (H). Horizontal line: mean; whiskers: 2×SEM interval. Data information: Statistical tests in (E–H): multiple all‐against‐control pairwise ANOVA comparisons with Benjamin––Hochberg correction. Control was WT genotype or non‐sprayed synaptosomes. In all statistical tests, *P < 0.05, **P < 0.01, and ***P < 0.001 after Benjamini–Hochberg correction.

The majority of SV are linked to other SVs via molecular bridges previously termed connectors (Landis *et al*, 1988; Hirokawa *et al*, 1989; Fernández‐Busnadiego *et al*, 2010; Zuber & Lučić, 2019). The function and composition of connectors are not clear yet. It was earlier proposed that connectors limit SV dispersion and allow SV mobilization for release. It is generally assumed that synapsin is involved in connector formation and may be one of its components. It has been suggested that connectors reduce SV mobility and maintain a locally high SV concentration in the pre‐synapse. The connectivity level of an individual SV might be one of the factors defining the pool to which the SV belongs. To shed some light on the role of connectors, we analyzed SV connectivity in our datasets. We focused our analysis on the SVs located at a distance from the AZ PM lower than 250 nm in synaptosomes and lower than 900 nm in neurons (Fig 6A–H). Furthermore, we defined six distance groups: proximal (0–45 nm), intermediate (45–75 nm), distal I (75–150 nm), distal II (150–250 nm), distal III (250–450 nm), and distal IV (450–900 nm) similarly to previous studies (Fernández‐Busnadiego *et al*, 2010, 2013).

In non‐sprayed synaptosomes datasets, approximately 70% of the proximal and intermediate SVs were connected to other vesicles. In distal I and II regions, this value rose to 84% (*P* < 0.05 χ^2^ test with Benjamini–Hochberg correction) and 87% (*P* < 0.001), respectively (Fig 6D). Similarly, the number of connectors per vesicle significantly increased from the proximal region (1.63 ± 0.17) to the distal I region (2.39 ± 0.10, *P* < 0.001; ANOVA test with Benjamini–Hochberg correction) and the distal II region (2.92 ± 0.10, *P* < 0.001, Fig 6B).

We then compared the number of connectors per SV between non‐sprayed synaptosomes and early‐fusion or late‐fusion synaptosomes. In the proximal group, there were significantly more connectors in the early‐fusion group than in the non‐sprayed group (1.63 ± 0.17 and 2.69 ± 0.43, *P* < 0.05; ANOVA test with Benjamini–Hochberg correction) and this number significantly dropped to 0.95 ± 0.18 in the late‐fusion group (*P* < 0.05, Fig 6B). Consistently, the number of connectors per non‐triple‐tethered proximal SV significantly increased from 1.64 ± 0.17 in the non‐sprayed group to 2.69 ± 0.54 in the early‐fusion group (*P* < 0.05 ANOVA test with Benjamini–Hochberg correction) and dropped to 0.91 ± 0.19 in the late‐fusion group (*P* < 0.05, Fig 6F). The number of connectors per triple‐tethered proximal SVs showed the same pattern, but these results did not reach significance. When considering non‐tethered versus tethered proximal SVs, we observed no significant difference in the number of connectors per SV between the experimental groups (Fig 6H). We compared the connected fraction of proximal SVs between non‐sprayed and sprayed synaptosomes and found no significant difference (Figs 6D and EV3D and F).

**Figure EV4:**
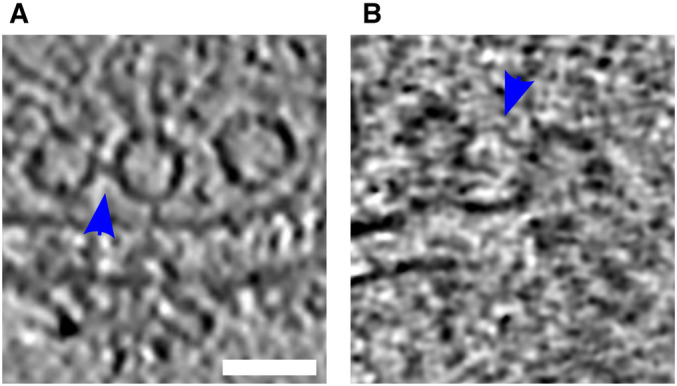
Tethered connected SVs A, B
Tomographic slices showing tethered connected vesicles. Blue arrows highlight the connectors. Scale bar, 50 nm.

Taken together, our observations indicate that following depolarization, the number of connectors per proximal SV with less than three tethers (i.e., non‐RRP) first increases and then decreases to a value lower than the initial one. We have seen earlier that the fraction of tethered proximal SVs does not differ between non‐sprayed and late‐fusion synaptosomes (Fig EV3B). Thus, our data suggest that establishing connectivity is a slower process than tethering. We hypothesize that given the free space made in the proximal region after some SVs have fused, vesicles from the intermediate region diffuse to the proximal zone and become tethered to the AZ PM, reaching non‐stimulated levels within the timeframe of our experiments. However, during this time, the number of connectors did not reach the non‐stimulated levels. Furthermore, we have observed that connectors remained present between fusing SV and neighbor SV (Fig EV4A and B). Thus, in addition to passive diffusion, pulling toward the plasma membrane of SV connected to fusing SV can contribute to replenishing the RRP.

We then analyzed SNAP‐25 neurons. For SNAP‐25‐WT, similarly to non‐sprayed synaptosomes, the fraction of connected SVs was significantly higher in the distal II and III regions than in the proximal region (*P* < 0.05 and *P* < 0.01, respectively, χ^2^ test with Benjamini–Hochberg correction), albeit the absolute values were overall lower than in synaptosomes (Fig 6C). Consistently, the number of connectors per SV in SNAP‐25‐WT synapses increased from 1.95 ± 0.38 in the proximal region to 3.23 ± 0.21 in the distal II region (Fig 6A, *P* < 0.05, ANOVA test with Benjamini–Hochberg correction). The fraction of connected SVs in the distal II region was significantly lower in the 4E and 4K mutants than in the WT (*P* < 0.05, χ^2^ test with Benjamini–Hochberg correction, Fig 6C). This was supported by a significantly lower number of connectors per SV in the distal II region for the 4E mutant versus the WT (*P* < 0.001, ANOVA test with Benjamini–Hochberg correction) as well as for the 4K mutant versus the WT (*P* < 0.001, Fig 6A). Furthermore, this number was also significantly lower in the distal III and IV regions for the 4E mutant versus the WT (*P* < 0.001). Consistently the fraction of connected SVs was lower in the distal II, III, and IV regions for the 4E mutant versus the WT (*P* < 0.05, *P* < 0.001, and *P* < 0.05, respectively, χ^2^ test with Benjamini–Hochberg correction, Fig 6C). The fraction of connected SVs was not different in the 4K mutant versus the WT, except in the distal IV region where it was higher (*P* < 0.05 χ^2^ test with Benjamini–Hochberg correction). The number of connectors per proximal SV and the connected fraction of proximal SVs were not affected by the mutations (Figs 6A, E and G, and EV3A, C and E). These results indicate that prolonged abnormal exocytotic activity, such as those caused by functional alterations of SNAP‐25, is correlated with severe changes in inter‐vesicular connectivity in the distal region.

## Discussion

Due to its transient nature, SV exocytosis has been difficult to characterize morphologically. A number of questions remain partially unresolved to this date. In particular, it has been suggested that following Ca^2+^ entry, the insertion of synaptotagmin‐1 into the membrane induces an increase in membrane curvature, which lowers the energy barrier of fusion. Such membrane deformations have been observed in biochemically reconstituted models of exocytosis but have not yet been reported in functional synapses (McMahon *et al*, 2010; Bharat *et al*, 2014). Moreover, it is not clear whether the membrane deformation occurs subsequently to Ca^2+^ influx or if primed SVs and their PM counterpart present such deformation (Bharat *et al*, 2014). The optimal sample preservation delivered by cryo‐ET makes it possible to investigate the role of tethers located between SVs and the AZ PM and the function of inter‐SV connectors. Combining cryo‐ET with spray‐mixing, plunge freezing enabled us to investigate the morphological changes occurring immediately after depolarization.

It should be noted that our study has some uncertainties and limitations. One uncertainty concerns the delay between stimulation and freezing. In future studies, this uncertainty could be reduced by stimulating exocytosis with a flash of light, on samples either expressing channelrhodopsin or containing caged calcium. Nonetheless, accessing sub‐millisecond delays, which is required to observe the early stages of SV exocytosis would be technically quite challenging with light stimulation. The identity of the proteins composing the tethers and connectors represents another uncertainty.

While stimulated synaptosomes were sprayed with a depolarizing solution, control synaptosomes were not sprayed. This may raise the concern that the observed changes are due the mere spraying and not depolarization. It should be noted that plunge freezing is typically done in an environment at 100% relative humidity to prevent sample drying. Consequently, water droplets likely also form on the sample surface when the sample is not sprayed. Furthermore, earlier studies have shown that the spray does not affect the near‐atomic structure of purified synaptic membranes (Unwin, 2005; Unwin & Fujiyoshi, 2012). It is, therefore, unlikely that the observed changes are due to the mere spraying. Another limitation is common to all cryo‐ET studies: to date, cytoplasmic protein labeling for cryo‐ET remains extremely challenging. Nevertheless, several laboratories, including ours, are working toward solving this limitation (preprint: Papantoniou *et al*, 2022). Finally, we could only analyze a restricted number of specimens. This was mainly due to two factors. Samples were relatively thick, and finding suitably thin synapses required extremely time‐consuming screening. In future studies, we might resort to cryo‐focused ion‐beam (cryo‐FIB) milling to prepare thin lamellas (Marko *et al*, 2007; Schaffer *et al*, 2017). Manual segmentation of SVs was possibly the most serious bottleneck. In the future, deep‐learning‐based segmentation procedures might reduce this burden.

### Membrane curvature increases following depolarization

Depolarization through spraying droplets of KCl solution on synaptosomes milliseconds before freezing allowed us to capture snapshots of exocytosis (Fig 3). In spite of the uncertainty on the exact delay between stimulation and freezing, our approach allowed access to shorter delays than any other cryo‐EM technique. Since the complete collapse of SVs occurs somewhere between 20 and 50 ms after stimulation (Heuser & Reese, 1981), we could observe early and late stages of exocytosis on the same spray‐mixed and plunge‐frozen synaptosome grid. The temporal sorting of observed exocytosis snapshots was done in the most parsimonious way. Nonetheless, it should be kept in mind that the annotation in early and late stages is a working hypothesis, which is supported by the literature (Heuser & Reese, 1981).

We observed that the curvature of some PM regions facing some SVs increased following depolarization. The SV facing such a PM buckling also seemed to get kinked. These deformations were not seen in non‐sprayed synaptosomes. This indicates that in functional synapses, exocytosis starts with a Ca^2+^‐dependent membrane deformation, which is supported by a wealth of *in vitro* biochemical data (McMahon *et al*, 2010; Rizo, 2022). Deformation may be caused in part by the intercalation of synaptotagmin‐1 C2A and C2B domains between membrane head groups. A recent biophysical study indicated that C2A and C2B preferably insert in SV membrane and PM, respectively (Nyenhuis *et al*, 2019). It may also be due to the tension/force induced by SNARE complex zippering (Gao *et al*, 2012). Subsequent snapshots showed a fuzzy contact point between the SV and the PM, which likely corresponds to lipid splaying or the merging of the two membranes. Membrane fusion then occurred and yielded classical Ω‐figures with variable neck diameters. Finally, nearly fully collapsed SVs were imaged. Overall our observations support the standard model of full‐collapse SNARE‐dependent membrane fusion (Risselada *et al*, 2011; Sharma & Lindau, 2018) and reveal details of exocytosis early stage, prior to actual membrane fusion.

### SV local concentration correlates with SV connectivity

Synaptic vesicle local concentration—a.k.a. SV occupancy—is tightly correlated with the distance from the AZ PM. Under resting conditions, SV occupancy showed a minimum in the intermediate region (45–75 nm away from the AZ) (Fig 4A and B), in agreement with previous reports (Fernández‐Busnadiego *et al*, 2010). By definition, all SVs in the proximal region are directly facing the PM. Their high concentration can be attributed to the fact that more than 50% of them are tethered to the PM. On the other hand, the number of connectors per SV and SV connectivity is generally higher in the distal regions (Fig 6A–D). This increased value correlates with higher SV occupancy. Thus, we may hypothesize that SV local concentration is a function of their level of tethering to the PM and of connection with other SVs. Interestingly, under short stimulation of a few milliseconds, SV occupancy only decreased in the proximal region, most likely as a consequence of the fusion of SVs with the PM (Fig 4B).

In order to further assess the relation between SV tethering, connectivity, and occupancy, we analyzed synapses expressing either WT SNAP‐25, a more positively charged mutant (4K), or a more negatively charged mutant (4E) (Ruiter *et al*, 2019). The 4K mutant has a decreased energy barrier to membrane fusion and causes constitutive active exocytosis, whereas the 4E mutant shows a decreased exocytotic activity because of a higher energy barrier to membrane fusion. Our data show that the 4K mutant had a significantly decreased proximal SV occupancy, while there was no significant difference in the case of the 4E mutant (Fig 4A). The decrease was probably due to the high frequency of spontaneous exocytosis observed in the 4K mutant (Ruiter *et al*, 2019). In the intermediate, and distal I regions, the occupancy of both mutants was very similar to the one of the WT. In more distal regions, the variability in occupancy between individual active zones strongly increased and made inter‐group comparisons difficult. Our data show that strong disturbances in exocytotic activity lead to profound differences in SV connectivity. We note that a correlation exists between SV connectivity and concentration. Future studies will be necessary to assess whether SV concentration depends on SV connectivity and to decipher the molecular mechanism influencing these parameters.

### SNAP‐25 4K mutant further supports the RRP morphological definition

Previously, we showed that the number of tethers of an SV defines whether its exocytosis can be induced by treatment with a hyperosmotic sucrose solution, which corresponds to a definition of the RRP (Fernández‐Busnadiego *et al*, 2010; Zuber & Lučić, 2019). We reported that SVs with at least three tethers belong to the RRP, according to this definition. In order to further assess this model, we analyzed synapses of neurons expressing the SNAP‐25 mutants. Seventeen percent of the WT proximal SVs had three tethers or more. Critically, the 4K mutant had no such SV. As the RRP (assessed with hyperosmotic sucrose treatment) in this mutant was formerly shown through functional assays to be depleted, our present observation further supports our morphological definition of the RRP (Ruiter *et al*, 2019). Fifteen per cent of the proximal SVs had three tethers or more in the 4E mutant, which is very similar to the WT situation, while this mutant was shown to possess a normal‐sized RRP. Our observations are also consistent with a number of studies that have concluded that SV exocytosis requires a minimum of three SNARE complexes (Domanska *et al*, 2009; Mohrmann *et al*, 2010; Shi *et al*, 2012).

### Depolarization rapidly induces additional tethering in proximal vesicles

**Figure 7:**
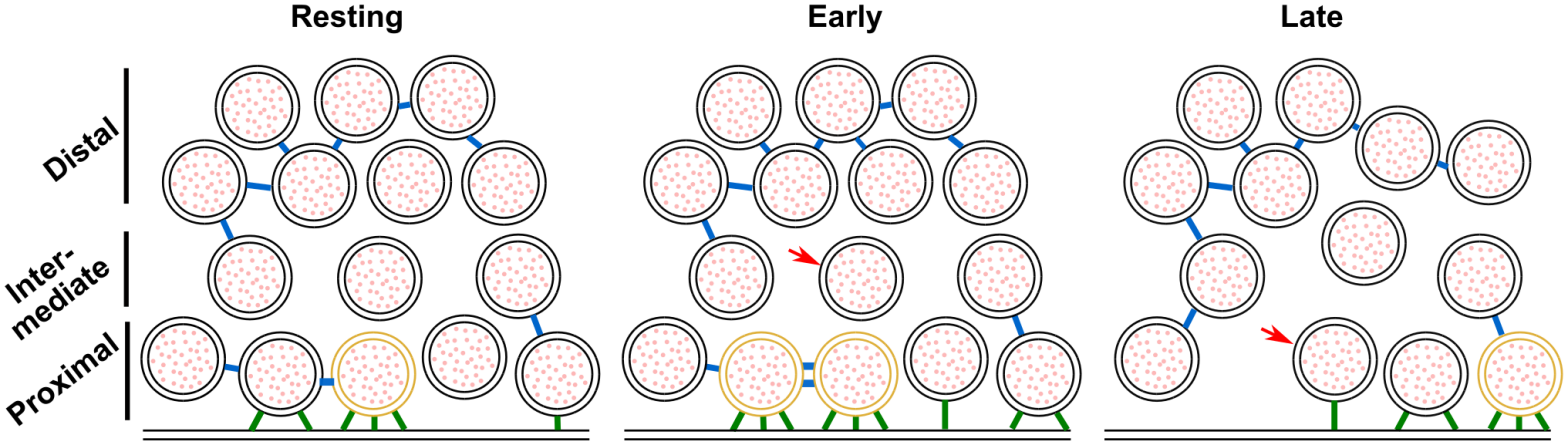
Model depicting a synapse transitioning from resting state to early‐ and late‐fusion states Tethering and connectivity changes upon synapse stimulation are depicted. Proximal non‐triple‐tethered vesicles (black proximal SVs) gain additional tethers, and some of them become triple tethered (yellow SVs) shortly after stimulation. Primed vesicles then fuse with the plasma membrane (late fusion) and leave an empty space in the AZ cytoplasm. The number of connectors (depicted in blue) per proximal SV decreases in late‐fusion tripled‐tethered vesicles. The red arrow shows a vesicle initially located in the intermediate region, which diffuses to the proximal region in the late‐fusion state. Tethers are shown in green.

We compared SV tethering before and shortly after depolarization. Our observations are schematically summarized in Fig 7. Interestingly, the fraction of proximal SVs that were tethered increased by 50% shortly after stimulation. Simultaneously, the number of tethers per proximal SV more than doubled, and the fraction of proximal SVs with three or more tethers tripled. All these measurements returned to pre‐stimulation values in pre‐synaptic terminals presenting more advanced stages of exocytosis (late‐fusion stage). These data indicate that immediately after the onset of stimulation, a quick and massive increase in tethering occurs. This phenomenon was resolved in our measurements because the spraying of synaptosomes with an intermediate K^+^‐concentration made it possible to detect synaptosomes in an early stage of fusion, which would have been missed during either strong or chronic stimulation because these would deplete primed vesicles.

The phenomenon of rapid, depolarization‐induced tethering leads to some free proximal SVs becoming tethered to the AZ PM, while some previously single‐ or double‐tethered SVs gained the additional tether(s) thus rendering them releasable according to our definition of the RRP (as triple‐tethered vesicles) (Fernández‐Busnadiego *et al*, 2010). There are several important implications of this finding. First, the increase in the number of tethers during the initial membrane contact—in excess of the three tethers formed during priming—might help overcome the fusion barrier. Functional reconstruction led to the suggestion that SNARE complexes primarily form downstream of Ca^2+^ influx (van den Bogaart *et al*, 2011), whereas mutagenesis studies in cells supported the notion that SNARE complexes had already formed before arrival of the Ca^2+^ trigger, that is, during priming (Walter *et al*, 2010). In fact, both notions might be partly correct, as the formation of a low number of SNARE complexes might lead to a stable primed state, defined by a valley in the energy landscape due to the dual inhibitory/stimulatory features of the SNARE complex (Weber *et al*, 2010; Ruiter *et al*, 2019), whereas more SNARE complexes might form dynamically after triggering, during membrane fusion itself. Accordingly, in *in vitro* fusion assays, additional SNARE complexes, in addition to those required for fusion pore formation, cause fusion pore stabilization (Shi *et al*, 2012; Bao *et al*, 2018). Second, vesicles that have not formed three tethers before stimulation might fuse with delayed kinetics during triggering, which accounts for the variable exocytosis kinetics among SVs (Lee *et al*, 2013; Neher, 2015; Taschenberger *et al*, 2016; Kobbersmed *et al*, 2020). Super‐primed vesicles are expected to have formed the largest number of tethers before stimulation (Lee *et al*, 2013; Taschenberger *et al*, 2016). Third, functionally overlapping protein complexes might be involved in priming and triggering, depending on the timing of their formation. Accordingly, triggering that stimulates tether formation might also stimulate priming for those vesicles that were not tethered before stimulation. Indeed, a number of recent publications have suggested that some SVs can get primed extremely quickly in response to Ca^2+^ influx (Miki *et al*, 2016; Schmidt, 2019; Kobbersmed *et al*, 2020; Kusick *et al*, 2020; Silva *et al*, 2021).

### Conclusion

Our study revealed fine morphological changes occurring in the pre‐synaptic terminal immediately after the onset of exocytosis, as well as in chronically active or inactive synapses. It indicates that the rise in pre‐synaptic Ca^2+^ induced increased SV tethering prior to SV fusion, which potentially corresponds to SV super‐priming. We also detected modifications of proximal SV interconnections in response to evoked exocytosis, as well as more drastic modifications of distal SV interconnections in chronically active synapses and in inactive synapses. These changes likely affect SV mobility and recruitment at the AZ.

## Materials and Methods

### Constructs and viruses

SNAP‐25B was N‐terminally fused to GFP and cloned into a pLenti construct with a CMV promoter (Delgado‐Martinez *et al*, 2007). Mutations were made using the QuikChange II XL kit (Agilent). The mutations were verified by sequencing and have been published before (Ruiter *et al*, 2019). The preparation of lentiviral particles followed standard protocols.

### Animals

Synaptosomes were prepared from adult male or female Wistar rats obtained from the central animal facilities of the Department of Biomedical Research of the University of Bern. Adult male or female Wistar rats at an age of 6–8 weeks were slightly stunned by CO_2_ and quickly decapitated with a guillotine. The procedures used were in accordance with the Swiss Veterinary Law guidelines. Heterozygous SNAP‐25 KO C57/Bl6 mice were routinely backcrossed to Bl6 to generate new heterozygotes. The strain was kept in the heterozygous condition, and timed heterozygous crosses and cesarean section were used to recover knockout embryos at embryonic day 18 (E18). Pregnant females were killed by cervical dislocation; embryos of either sex were collected and killed by decapitation. Permission to keep and breed SNAP‐25 mice was obtained from the Danish Animal Experiments Inspectorate, and institutional guidelines were followed as overseen by the Institutional Animal Care and Use Committee (IACUC). Newborn (P0‐P2) CD1 outbred mice of either sex were used to create astrocytic cultures and therefore were killed by decapitation.

### Synaptosome preparation

Rat synaptosomes were prepared as previously described (Dunkley *et al*, 2008) with some modifications. The cerebral cortex and the hippocampi were removed in sucrose buffer (SEH: 0.32 M sucrose, 1 mM EDTA, and 10 mM HEPES; HEPES, #H4034, Sigma‐Aldrich Corporate Offices. St. Louis, MO, USA) on ice. Homogenization of the tissue was done in SEH with a Potter‐Elvehjem grinder (#358011, Wheaton, Millville, New Jersey, USA), four strokes at the bottom and six from top to bottom were applied to the tissue at a speed of 800 turns/min as described in Dunkley *et al* (2008). The whole process from decapitation to homogenization was done within 2–3 min to obtain functional synaptosomes. Homogenized tissue was then centrifuged at 1,000 *g* for 10 min at 4°C to remove meninges and blood vessels. The resulting supernatant containing not only synaptosomes but also gliosomes and mitochondria was then added to a discontinuous, isosmotic Percoll (#P1644, Sigma) gradient with 5, 10, and 23% in 0.32 M sucrose and 1 mM EDTA in centrifuge tubes (#344060, Beckman Coulter). The samples were spun in an ultracentrifuge (rotor: SW 40 Ti; Beckman Coulter, Nyon, Switzerland) at 47,000 *g* for 12 min at 4°C. The layer with the highest amount of functional synaptosomes was between 10 and 23% (Dunkley *et al*, 2008). The layer was carefully taken out and diluted 1:10 in HEPES‐buffered medium (HBM; 140 mM NaCl, 5 mM KCl, 5 mM NaHCO_3_, 1.2 mM Na_2_HPO_4_, 1 mM MgCl_2_, 10 mM Glucose, and 20 mM HEPES). The obtained solution was further spun with an ultracentrifuge (rotor 45 Ti; Beckman Coulter) at 14,540 *g* for 20 min at 4°C. The pellet was carefully and quickly aspirated with a Pasteur pipette to avoid mixture with the solution and then diluted in HBM.

### Preparation of astrocytic and neuronal culture

Glial cells were prepared as described previously (Shahmoradian *et al*, 2014). Astrocytes were isolated from CD1 outbred mice (P0–P2). Pups were killed by decapitation and heads were placed in HBSS‐HEPES medium (HBSS supplemented with 1 M HEPES). The cortices were isolated from the brains and the meninges were removed (dura, pia, and arachnoid mater). The cortices were chopped into smaller fragments and transferred to a tube containing 0.25% trypsin dissolved in Dulbecco's modified Eagle media (DMEM). Fragments were incubated for 15 min at 37°C. Subsequently, inactivation medium (12.5 mg albumin + 12.5 mg trypsin inhibitor in DMEM + 10% FBS) was added and the tissue was washed with HBSS‐HEPES. Tissue was triturated until a smooth, cloudy suspension appeared. Cells were plated in 75 cm^2^ flasks with pre‐warmed DMEM, one hemisphere per flask, and stored at 37°C with 5% CO_2_. Glial cells were ready to be used after 10 days. Glial cells were washed with pre‐warmed HBSS‐HEPES. Trypsin was added and the flasks were incubated at 37°C for 10 min. Cells were triturated and counted with a Buerker chamber before plating 100,000 cells/ml on untreated 12‐well plates containing DMEM supplemented with 10% fetal bovine serum (FBS), 10,000 IU penicillin, 10 mg streptomycin, and 1% DMEM non‐essential amino acids. After 2 days, neurons were plated.

Hippocampal neurons were isolated from E18 SNAP‐25 KO of either sex. The SNAP‐25 KO pups were obtained by pairing two heterozygote animals, and the embryos were recovered at E18 by cesarean section. Pups were selected based on the absence of motion after tactile stimulation and bloated neck (Washbourne *et al*, 2001); the genotype was confirmed by PCR in all cases. The pups were killed by decapitation and heads were put in HBSS‐HEPES medium. The cortices were isolated from the brains and the meninges were removed. The hippocampi were cut from the cortices before being transferred to a tube containing 0.25% trypsin dissolved in HBSS‐HEPES solution. Fragments were incubated for 20 min at 37°C. Afterward, the tissue was washed with HBSS‐HEPES. The hippocampi were triturated and the cell count was determined with a Buerker chamber. Twenty microliter of solution containing 250,000 cells/ml were plated onto the flame‐sterilized gold R2/2 or R2/1 EM grids as previously described in Shahmoradian *et al* (2014). Following a 30‐min incubation at 37°C, the grid was transferred into the 12‐well plate containing the astrocytes, and medium was replaced with NB medium (neurobasal with 2% B‐27, 1 M HEPES, 0.26% lutamax, 14.3 mM β‐mercaptoethanol, 10,000 IU penicillin, and 10 mg streptomycin) for the E18 pups. Between 4 h and 1 day later, lentiviral particles carrying SNAP‐25‐WT, SNAP‐25‐4E, or SNAP‐25‐4K constructs were added to the culture (Ruiter *et al*, 2019). The cultures were incubated for 12–14 days before being plunge frozen.

### Plunge freezing and spray mixing

Rat synaptosomes were prepared for plunge freezing and spray mixing as follows. The following steps from incubation to plunge freezing were all done at room temperature (RT), equivalent to 23–25°C. The synaptosomal solution was incubated with calcein blue AM (#C1429, Molecular Probes—Thermo Fisher Scientific, Waltham, MA, USA) 30 min prior to plunge freezing to visualize the cytosol of functional—esterase containing—cellular compartments such as synaptosomes. Additionally, 1.3 mM CaCl_2_ and 10 nm gold fiducials were added (gold fiducials, #s10110/8, AURION Immuno Gold Reagents & Accessories, Wageningen, The Netherlands). From the synaptosome suspension obtained from an animal, some was used for control experiments (plunge frozen without spray mixing) and some was stimulated (spray mixed and plunge frozen). CaCl_2_ is necessary to trigger exocytosis, and gold fiducials are important to align the acquired tilt series for tomogram reconstruction. The sprayed solution contained 1 mM CaCl_2_ and 52 mM KCl in HBM to depolarize synaptosomes and trigger exocytosis. It also contained fluorescein (#46955, Sigma) to trace the spray droplets on the EM grid in cryo‐FM. The synaptosomal solution was applied to a 200‐mesh lacey finder carbon film grid (#AGS166‐H2. Agar Scientific. Elektron Technology UK Ltd, Stansted, UK). Excess liquid on the grid was removed by blotting with a filter paper and the grid was immediately plunge frozen in liquid ethane with a home‐built plunge freezer and was sprayed on the fly. The plunge freezer and the spraying device (atomizer) were computer controlled with a LabView script (National Instruments Corporation, Mopac Expwy Austin, TX, USA). The spraying device was set similarly to the device in Berriman and Unwin (1994). Nitrogen gas pressure necessary to drive spraying was set to 2.5 bar. The grid was set to pass in front of the spray nozzle at a distance of 3–4 mm. The plunge freezer was accelerated to 0.75 m/s and the height of the spray nozzle was adjusted so that the minimum spray delay was ~7 ms. The delay could be adjusted by varying the distance between the spray nozzle and the cryogen bath, and the maximum delay that we used was 35 ms. The atomizer sprays scattered droplets of various sizes on the EM grid. During the time lapse between spraying and freezing the content of the droplets spreads by diffusion. KCl diffuses approximately 4× faster than fluorescein. Cryo‐ET imaging was done within the diffusion distance of KCl but outside of the visible spray droplet because the center of the spray droplet would usually be too thick for imaging. This allows reaching minimal stimulation times of few milliseconds or even sub‐milliseconds and maximal stimulation times of 35 ms. Moreover, through diffusion, KCl concentration rapidly rises and then decreases. Hence, synaptosomes are not permanently depolarized.

After 12–14 days of incubation, grids with mouse neurons were plunge frozen with a Vitrobot (Thermo Fisher Scientific, Mark IV) with a blot time of 3 s and a blot force of −10. Wait time and drain time were not used. Humidity was set to 100% at 4°C. Four microliter undiluted 10 nm BSA gold tracer (Aurion) was added directly onto the grid prior to plunge freezing.

### Cryo‐fluorescence microscopy

After plunge freezing, rat synaptosome samples were imaged at the fluorescent microscope under cryo‐conditions, with a Zeiss Axio Scope.A1, equipped with an AxioCam MRm camera (Carl Zeiss AG, Germany) and a fluorescence lamp (HXP 120 C). The correlative microscopy stage (#CMS196, Linkam Scientific Instruments, UK) was cooled down to −190°C by liquid nitrogen, and the frozen EM grid was placed into the chamber of the cryo‐stage on a bridge that was partially submerged in liquid nitrogen and was close to the objective, where the temperature was around −150°C. The filter set used for imaging fluorescein was #38 (#000000‐1031‐346, Zeiss) (BP 470/40, FT 495, BP 525/50; corresponds to GFP) and the one for calcein blue AM was #49 (#488049‐9901‐000, Zeiss) (G 365, FT 395, BP 445/50; corresponds to DAPI). The objective used was either a 10× (#420941‐9911, NA = 0.25 Ph1, Zeiss) or a 50× (#422472‐9900, NA = 0.55 Dic, Zeiss), the acquisition software used was AxioVision (AxioVs40x64 V 4.8.3.0, Zeiss) and the processing software was ZEN lite (Zeiss).

### Cryo‐electron microscopy

Following cryo‐FM, the rat synaptosome grids were mounted in a cryo‐holder (Gatan, Pleasonton, CA, USA) and transferred to a Tecnai F20 (FEI, Eindhoven, The Netherlands), which was set to low‐dose conditions, operated at 200 kV, and equipped with a field emission gun. Images were recorded with a 2k × 2k CCD camera (Gatan) mounted after a GIF Tridiem post‐column filter (Gatan) operated in zero‐loss mode. The sample was kept at about −180°C. Tilt series were acquired using SerialEM (Mastronarde, 2005) for automated acquisition recorded typically from −50° to 50° with a 2° angular increment and an unbinned pixel size of 0.75 or 1.2 nm. Due to sample thickness (400–700 nm), tomograms were usually not recorded with higher tilt angles. Defocus was set between −8 and −12 μm and the total electron dose used was about 80–100 e^−^/Å^2^. Some tomograms were acquired at a Titan Krios equipped with a K2 direct electron detector (Gatan) without energy filter. The K2 camera was operated in super‐resolution counting mode, and between 8 and 40 frames per tilt angle were taken. Tilt series were acquired using the Latitude software (Gatan) for automated acquisition recorded typically from −60° to 60° with a 2° angular increment and an unbinned pixel size of 0.6 nm. Defocus was set between −8 and −12 μm and the total electron dose used was about 80–100 e^−^/Å^2^. Prior to image processing the frames at each tilt angle, frames were aligned and averaged in 2dx MC_Automator (Scherer *et al*, 2014) with motioncor (Li *et al*, 2013). 3D reconstruction was done in IMOD (Kremer *et al*, 1996). The alignments were done using the automated fiducial tracking function, and the 3D reconstructions were done using the weighted back projection followed by a nonlinear anisotropic diffusion (NAD) filtering. Following tomogram, reconstruction‐only synaptosomes that fulfilled the following criteria were used: (i) even and non‐broken PM, (ii) post‐synaptic membrane still attached to the pre‐synapse, (iii) spherical vesicles, and (iv) a mitochondrion in the pre‐synapse necessary to cover the energy demands of the synapse. These criteria indicate that the synaptosome is functional (Harrison *et al*, 1988). The criteria were decided before the experiments and were applied to all synaptosomes in the dataset.

Cultured mouse neuron tilt series were acquired at a Titan Krios, equipped with a Falcon 3 direct electron detector (Thermo Fisher Scientific) without energy filter. The Falcon camera was operated in linear mode. Tilt series were acquired using the TEM Tomography software (TFS) for automated acquisition recorded typically from −60° to 60° with a 2° angular increment and an unbinned pixel size of 0.37 nm. Defocus was set between −6 and −10 μm and the total electron dose used was about 80–100 e^−^/Å^2^. Tomogram reconstruction was done for synaptosome datasets. The same inclusion/exclusion criteria as for synaptosome datasets were applied to mouse neuron datasets.

### Manual and automatic segmentation procedures

Manual segmentation of SVs, mitochondria, and the active‐zone PM was done in IMOD (Fig EV1B and D, Movies EV1 and EV2). The boundary marked the region to be analyzed by Pyto (Lučić *et al*, 2016). The analysis by Pyto was essentially the same as described previously (Fernández‐Busnadiego *et al*, 2010; Lučić *et al*, 2016). In short, the segmented area is divided into 1‐voxel‐thick layers parallel to the active zone for distance calculations. A hierarchical connectivity segmentation detects densities interconnecting vesicles (so‐called connectors) and densities connecting vesicles to the active‐zone PM (so‐called tethers) (Fig EV1B and D, Movies EV1 and EV2). Distance calculations are done with the center of the vesicle. Mainly default settings were used. The segmentation procedure is conservative and tends to miss some tethers and connectors because of noise. Consequently, the numbers of tethers and connectors should not be considered as absolute values but rather to compare experimental groups. All tomograms analyzed by Pyto were obtained on the same microscope with the same tilt range. The margin of error for false negatives and positives was found to be < 10% by comparison with ground truth (Lučić *et al*, 2016). As was done before, an upper limit was set between 2,100 and 3,200 nm^3^ on segment volume. The tomograms that were used for this analysis were binned by a factor of 2–3, resulting in voxel sizes between 2.1 and 2.4 nm. Tether and connector length was calculated using the midpoint method (Lučić *et al*, 2016). From the stimulated synaptosomes, only those that showed visible signs of exocytosis were used for analysis in Pyto.

### Data analysis

No blinding of the data was performed. If not stated otherwise, data in the text are described as mean ± standard error of the mean (SEM). Statistical tests are described in figure legends. Multiple pairwise χ^2^ test with Benjamini–Hochberg correction was used to compare data in form of a fraction of SVs pooled from all active zones of an experimental group (e.g., in Fig 5A and B) (Benjamini & Hochberg, 1995). Multiple pairwise ANOVA comparisons with Benjamini–Hochberg correction were used in all other cases. All‐against‐reference pairwise comparisons were performed. The definition of references is described for each comparison in the corresponding figure legend. We did not apply statistical methods to predetermine sample size but similar sample sizes as previously reported have been used (Fernández‐Busnadiego *et al*, 2010). It was not necessary to apply randomization. We performed Benjamini–Hochberg correction with the *multipletests* function implemented in the Python module *statsmodels* (Seabold & Perktold, 2010). A list of *P*‐values resulting from pairwise comparisons was input, and *multipletests* output was a list of corrected *P*‐values. The used implementation of the Benjamini–Hochberg correction does not require a false discovery rate to be input. This variation of the original Benjamini–Hochberg correction algorithm was proposed by Yekutieli and Benjamini (1999). If a corrected *P*‐value is smaller than the defined acceptable false discovery rate, then the null hypothesis is rejected, that is, the difference is considered statistically significant. This algorithm enables to test multiple false discovery rates in one step and its conclusions are exactly the same as the original Benjamini–Hochberg correction algorithm run multiple times with different false discovery rates. In the figures, ***, **, and * indicate a corrected *P*‐value lower than false discovery rates of 0.001, 0.01, and 0.05, respectively.

### Manuscript preparation

The manuscript was written with the open and collaborative scientific writing package Manubot (Himmelstein *et al*, 2019). The source code and data for this manuscript are available at https://github.com/aseedb/synaptic_tomo_ms.

## Author contributions

**Julika Radecke:** Conceptualization; data curation; funding acquisition; investigation; writing – original draft; writing – review and editing. **Raphaela Seeger:** Conceptualization; data curation; investigation; writing – original draft; writing – review and editing. **Anna Kádková:** Investigation; writing – review and editing. **Ulrike Laugks:** Investigation; writing – review and editing. **Amin Khosrozadeh:** Investigation; writing – review and editing. **Kenneth N Goldie:** Investigation; writing – review and editing. **Vladan Lučić:** Data curation; investigation; writing – review and editing. **Jakob B Sørensen:** Conceptualization; supervision; funding acquisition; writing – original draft; project administration; writing – review and editing. **Benoît Zuber:** Conceptualization; data curation; supervision; funding acquisition; writing – original draft; project administration; writing – review and editing.

In addition to the CRediT author contributions listed above, the contributions in detail are:

JR, RS, JBS, and BZ designed the study. JR, RS, and Anna K performed the experiments. KG provided access to and assistance with one of the Titan Krios microscopes. JR, RS, and BZ analyzed the data. VL, UL, and Amin K contributed to the Pyto analysis. JR, RS, JBS, and BZ wrote the manuscript with contributions from all authors. JBS and BZ supervised the project. Open access funding provided by Universitat Bern.

## Disclosure and competing interests statement

The authors declare that they have no conflict of interest.

## Supporting information



Expanded View Figures PDF

Table EV1

Table EV2

Movie EV1

Movie EV2

PDF+

## Data Availability

This study includes no data deposited in external repositories.
