# Supplementary material for: Morphofunctional changes at the active zone during synaptic vesicle exocytosis
Source: EMBO Rep. 2023 Mar 6;24(5):e55719. doi: 10.15252/embr.202255719 (PMC10157379; doi:10.15252/embr.202255719)
Supplement: Supplementary file 1 — Expanded View Figures PDF [file EMBR-24-e55719-s001.pdf]

Expanded View Figures

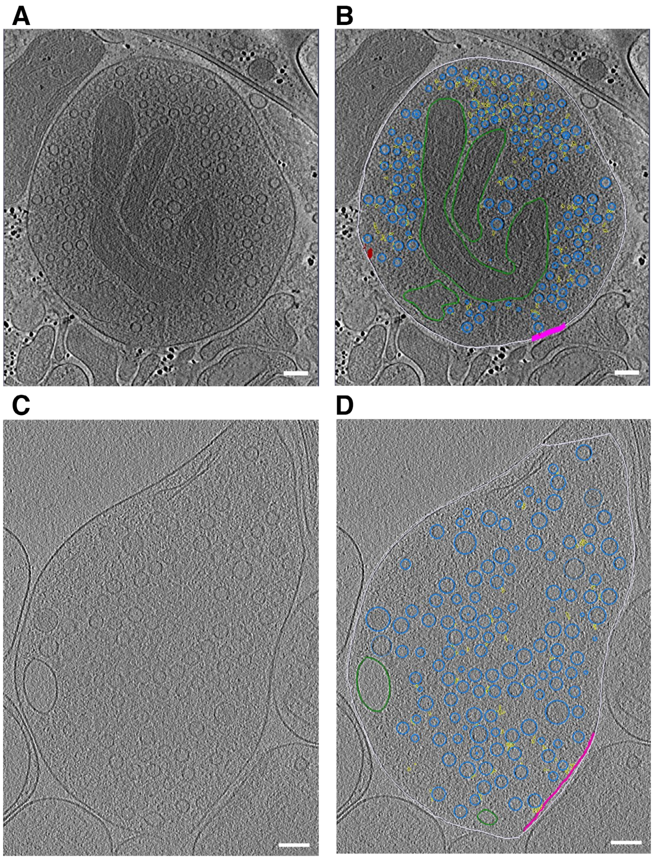

Figure EV1. Representative slices through tomograms.

A, B Tomographic slice without (A) and with (B) segmentation of synaptosome with late-fusion events.

C, D Tomographic slice without (C) and with (D) segmentation of WT SNAP-25 neurons.

Data information: segmentation colors: off-white = cell outline; pink = active zone; blue = synaptic vesicles; green = mitochondria; yellow = connectors and red = tethers. Scale bar, 100 nm.

© 2023 The Authors

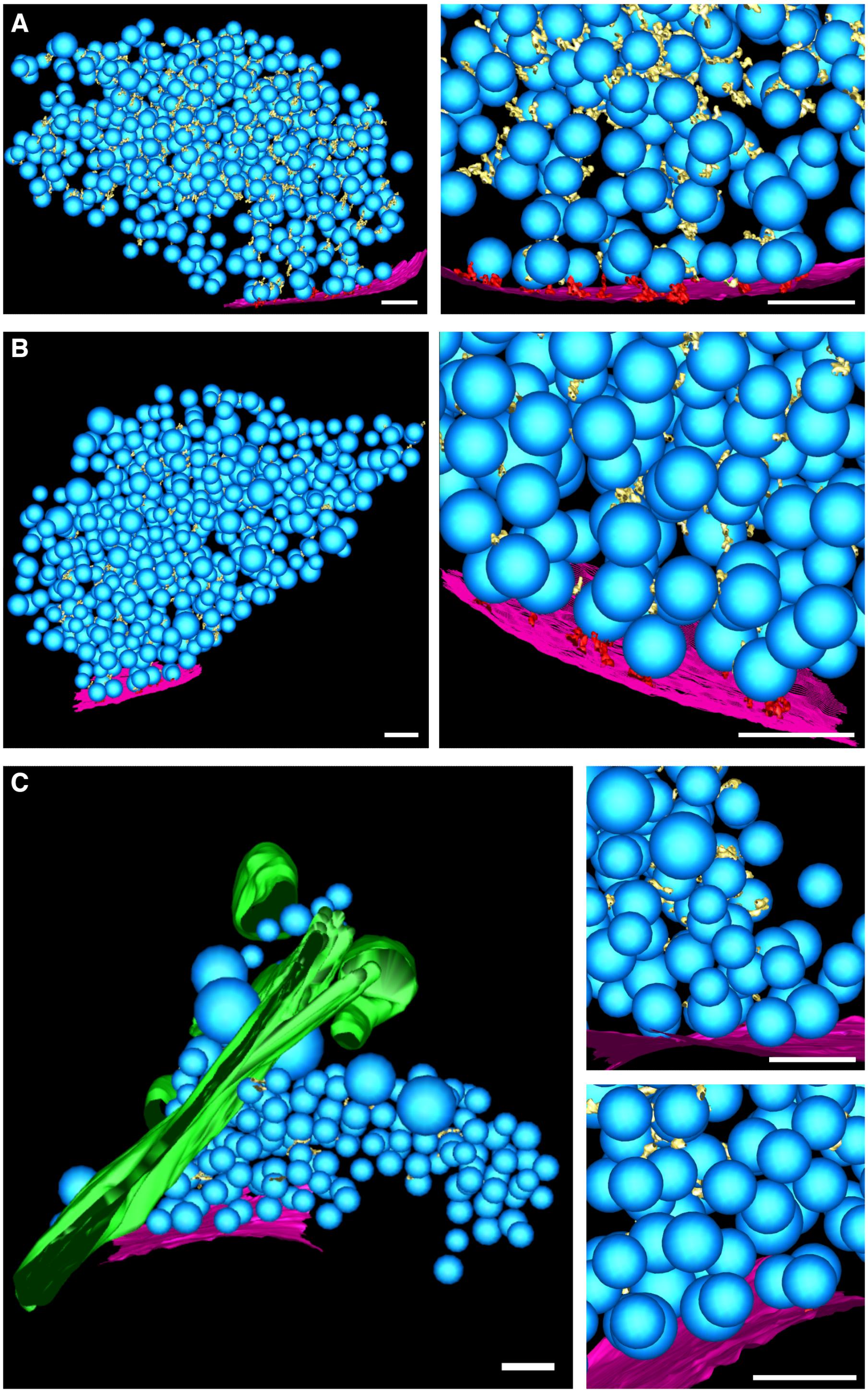

Figure EV2. 3-D rendered segmented tomograms of neuron synapses.

A–C (A) SNAP-25 WT, (B) SNAP-25-4E, and (C) SNAP-25-4K. (left) Overview, (right) detail. Blue: synaptic vesicles; purple: active-zone plasma membrane; green: endoplasmic reticulum-like organelle; yellow: connectors; and red: tethers. Scale bars: 100 nm.

 © 2023 The Authors

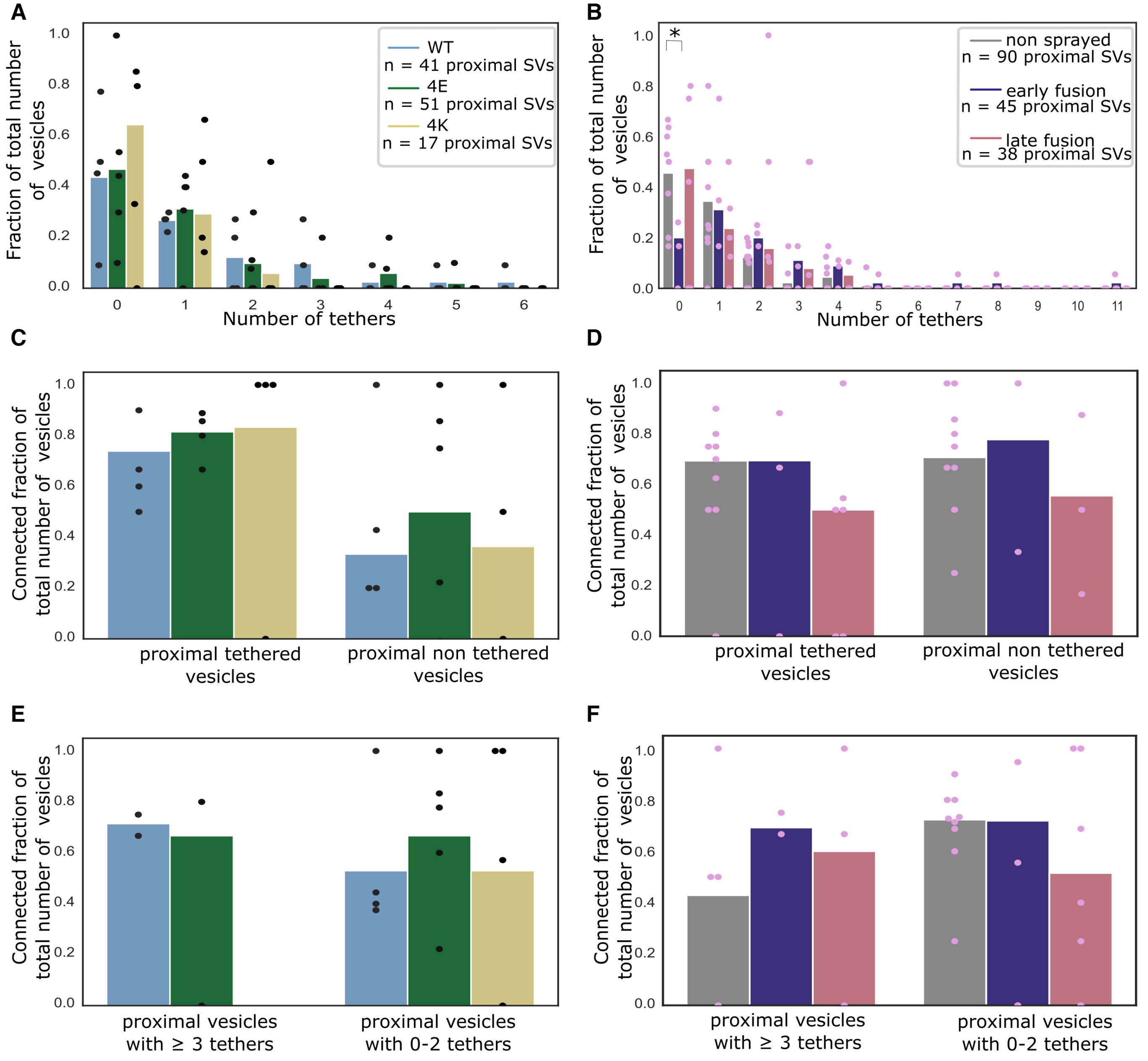

Figure EV3. Additional SV tethering and connectivity data.

A, B Histogram of the number of tethers per proximal SV. Statistical test: pairwise χ^2 test between control and each experimental condition in the zero-tether group with Benjamini–Hochberg correction. *$P < 0.05$.

C, D Histogram of connected SV among tethered or non-tethered proximal SVs.

E, F Histogram of connected SV among proximal non-RRP or RRP SVs.

Data information: (A, C, E) Synapses in mouse cultured neurons. (B, D, F) Rat synaptosomes.

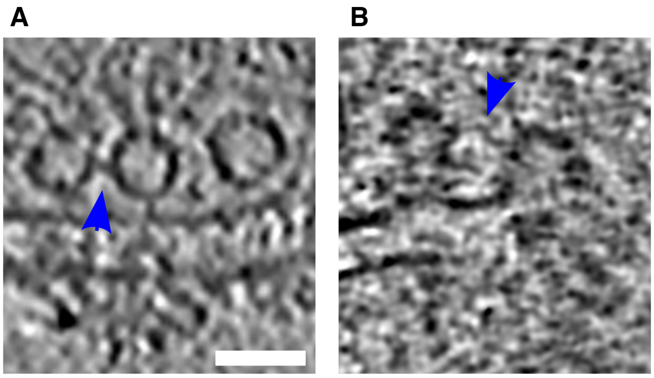

Figure EV4. Tethered connected SVs.

A, B Tomographic slices showing tethered connected vesicles. Blue arrows highlight the connectors. Scale bar, 50 nm.

 © 2023 The Authors
