# Supplementary material for: Morphofunctional changes at the active zone during synaptic vesicle exocytosis
Source: EMBO Rep. 2023 Mar 6;24(5):e55719. doi: 10.15252/embr.202255719 (PMC10157379; doi:10.15252/embr.202255719)
Supplement: Supplementary file 2 — Table EV1 [file EMBR-24-e55719-s006.docx]

Table EV1: Summary of the synaptosome tomograms.

| ID | Exocytosis stage | Vesicles per tomogram | Tethers per AZ | AZ surface area [µm²] | Connectors per synapse (0-250 nm) |
| --- | --- | --- | --- | --- | --- |
| Control 1 | not stimulated | 220 | 15 | 0.11 | 331 |
| Control 2 | not stimulated | 104 | 8 | 0.04 | 264 |
| Control 3 | not stimulated | 127 | 4 | 0.05 | 230 |
| Control 4 | not stimulated | 143 | 3 | 0.03 | 482 |
| Control 5 | not stimulated | 213 | 12 | 0.11 | 361 |
| Control 6 | not stimulated | 104 | 7 | 0.06 | 199 |
| Control 7 | not stimulated | 184 | 9 | 0.03 | 360 |
| Control 8 | not stimulated | 132 | 19 | 0.09 | 226 |
| Control 9 | not stimulated | 134 | 6 | 0.08 | 326 |
| Spray 1 | late | 697 | 5 | 0.03 | 272 |
| Spray 2 | late | 115 | 3 | 0.08 | 88 |
| Spray 3 | late | 429 | 21 | 0.19 | 882 |
| Spray 5 | early | 534 | 57 | 0.07 | 1412 |
| Spray 5_2 (second AZ, same synaptosome) | early |  | 32 | 0.15 |  |
| Spray 6 | late | 371 | 1 | 0.03 | 397 |
| Spray 7 | early | 107 | 5 | 0.02 | 156 |
| Spray 8 | late | 99 | 4 | 0.02 | 202 |
| Spray 10 | late | 76 | 4 | 0.02 | 96 |
