# Supplementary material for: Morphofunctional changes at the active zone during synaptic vesicle exocytosis
Source: EMBO Rep. 2023 Mar 6;24(5):e55719. doi: 10.15252/embr.202255719 (PMC10157379; doi:10.15252/embr.202255719)
Supplement: Supplementary file 3 — Table EV2 [file EMBR-24-e55719-s007.docx]

Table EV2: Summary of the neuron tomograms.

| ID | Mutation | Vesicles per Tomogram | Tethers per AZ | AZ surface area [µm²] | Connectors per synapse (0-250 nm) |
| --- | --- | --- | --- | --- | --- |
| 73 | 4E | 0-459 | 23 | 0.23 | 269 |
| 80 | 4E | 105 | 6 | 0.21 | 84 |
| 84 | 4E | 109 | 10 | 0.12 | 107 |
| 88 | 4E | 154 | 10 | 0.13 | 159 |
| 102 | 4E | 103 | 0 | 0.29 | 94 |
| 114 | 4K | 123 | 2 | 0.09 | 68 |
| 115 | 4K | 137 | 1 | 0.26 | 70 |
| 116 | 4K | 278 | 1 | 0.13 | 154 |
| 123 | 4K | 55 | 3 | 0.09 | 52 |
| 128 | WT-KO | 243 | 12 | 0.18 | 683 |
| 132 | WT-KO | 126 | 2 | 0.24 | 110 |
| 133 | WT-KO | 505 | 27 | 0.17 | 229 |
| 134 | WT-KO | 600 | 7 | 0.34 | 144 |
