# Supplementary material for: Morphofunctional changes at the active zone during synaptic vesicle exocytosis
Source: EMBO Rep. 2023 Mar 6;24(5):e55719. doi: 10.15252/embr.202255719 (PMC10157379; doi:10.15252/embr.202255719)
Supplement: Supplementary file 4 — Movie EV1 [file EMBR-24-e55719-s003.zip › Movie EV1 legend.docx]

**Movie EV1: Tomogram with segmentation of synaptosome with late fusion events.** off-white = cell outline; pink = active zone; blue = synaptic vesicles; dark green = mitochondria; light green = large vesicles; yellow = connectors, red = tethers, scale bar 100nm
