# Supplementary material for: Morphofunctional changes at the active zone during synaptic vesicle exocytosis
Source: EMBO Rep. 2023 Mar 6;24(5):e55719. doi: 10.15252/embr.202255719 (PMC10157379; doi:10.15252/embr.202255719)
Supplement: Supplementary file 7 [file EMBR-24-e55719-s004.pdf]

Morphofunctional changes at the active zone during synaptic vesicle exocytosis

Julika Radecke, Raphaela Seeger, Anna Kádková, Ulrike Laugks, Amin Khosrozadeh, Kenneth Goldie, Vladan Lucic, Jakob Sorensen, and Benoît Zuber
DOI: 10.15252/embr.202255719

Corresponding author(s): Benoît Zuber (benoit.zuber@unibe.ch) , Jakob Sorensen (jakobbs@sund.ku.dk)

Review Timeline:

Submission Date:	6th Jul 22
Editorial Decision:	22nd Aug 22
Revision Received:	8th Dec 22
Editorial Decision:	13th Jan 23
Revision Received:	30th Jan 23
Accepted:	16th Feb 23

Editor: Esther Schnapp

Transaction Report: This manuscript was transferred to EMBO reports following peer review at The EMBO Journal.

(Note: With the exception of the correction of typographical or spelling errors that could be a source of ambiguity, letters and reports are not edited. Depending on transfer agreements, referee reports obtained elsewhere may or may not be included in this compilation. Referee reports are anonymous unless the Referee chooses to sign their reports.)

1st Editorial Decision 22nd Aug 2022

Dear Benoit,

Thank you for the submission of your revised manuscript to EMBO reports.
We have now received the comments from both referees and from one advisor, as well as several cross-comments, all included below.

As you will see, referees 1 and 2 are not in agreement, and this is why I contacted an advisor/arbitrator. Upon cross-commenting, referee 1 also agrees with referee 2's concerns on the overall procedure and its limitations. However, both referee 1 and the advisor still support the publication of your study if you can provide more data-based support for your conclusions, and openly discuss the method, your assumptions and the limitations of the study.

I would thus like to invite you to revise your manuscript with the understanding that the referee concerns must be fully addressed and their suggestions taken on board. Please address all referee concerns in a complete point-by-point response. Acceptance of the manuscript will depend on a positive outcome of a second round of review. It is EMBO reports policy to allow a single round of major revision only and acceptance or rejection of the manuscript will therefore depend on the completeness of your responses included in the next, final version of the manuscript.

We realize that it is difficult to revise to a specific deadline. In the interest of protecting the conceptual advance provided by the work, we recommend a revision within 3 months (22nd Nov 2022). Please discuss the revision progress ahead of this time with the editor if you require more time to complete the revisions.

You can either publish the study as a short report or as a full article. For short reports, the revised manuscript should not exceed 27,000 characters (including spaces but excluding materials & methods and references) and 5 main plus 5 expanded view figures. The results and discussion sections must further be combined, which will help to shorten the manuscript text by eliminating some redundancy that is inevitable when discussing the same experiments twice. For a normal article there are no length limitations, but it should have more than 5 main figures and the results and discussion sections must be separate. In both cases, the entire materials and methods must be included in the main manuscript file.

IMPORTANT NOTE: we perform an initial quality control of all revised manuscripts before re-review. Your manuscript will FAIL this control and the handling will be DELAYED if the following APPLIES:
1) A data availability section providing access to data deposited in public databases is missing. If you have not deposited any data, please add a sentence to the data availability section that explains that.
2) Your manuscript contains statistics and error bars based on n=2. Please use scatter blots in these cases. No statistics should be calculated if n=2.

When submitting your revised manuscript, please carefully review the instructions that follow below. Failure to include requested items will delay the evaluation of your revision.

1) a .docx formatted version of the manuscript text (including legends for main figures, EV figures and tables). Please make sure that the changes are highlighted to be clearly visible.

2) individual production quality figure files as .eps, .tif, .jpg (one file per figure). See https://wol-prod-cdn.literatumonline.com/pb-assets/embo-site/EMBOPress_Figure_Guidelines_061115-1561436025777.pdf for more info on how to prepare your figures.

3) We replaced Supplementary Information with Expanded View (EV) Figures and Tables that are collapsible/expandable online. A maximum of 5 EV Figures can be typeset. EV Figures should be cited as 'Figure EV1, Figure EV2" etc... in the text and their respective legends should be included in the main text after the legends of regular figures.

- For the figures that you do NOT wish to display as Expanded View figures, they should be bundled together with their legends in a single PDF file called *Appendix*, which should start with a short Table of Content. Appendix figures should be referred to in the main text as: "Appendix Figure S1, Appendix Figure S2" etc. See detailed instructions regarding expanded view here: <https://www.embopress.org/page/journal/14693178/authorguide#expandedview>

- Additional Tables/Datasets should be labeled and referred to as Table EV1, Dataset EV1, etc. Legends have to be provided in a separate tab in case of .xls files. Alternatively, the legend can be supplied as a separate text file (README) and zipped together with the Table/Dataset file.

4) a .docx formatted letter INCLUDING the reviewers' reports and your detailed point-by-point responses to their comments. As part of the EMBO Press transparent editorial process, the point-by-point response is part of the Review Process File (RPF), which will be published alongside your paper.

5) a complete author checklist, which you can download from our author guidelines

<https://www.embopress.org/page/journal/14693178/authorguide>. Please insert information in the checklist that is also reflected in the manuscript. The completed author checklist will also be part of the RPF.

6) Please note that all corresponding authors are required to supply an ORCID ID for their name upon submission of a revised manuscript (<https://orcid.org/>). Please find instructions on how to link your ORCID ID to your account in our manuscript tracking system in our Author guidelines
<https://www.embopress.org/page/journal/14693178/authorguide#authorshipguidelines>

7) Before submitting your revision, primary datasets produced in this study need to be deposited in an appropriate public database (see https://www.embopress.org/page/journal/14693178/authorguide#datadeposition). Please remember to provide a reviewer password if the datasets are not yet public. The accession numbers and database should be listed in a formal "Data Availability" section placed after Materials & Method (see also https://www.embopress.org/page/journal/14693178/authorguide#datadeposition). Please note that the Data Availability Section is restricted to new primary data that are part of this study. * Note - All links should resolve to a page where the data can be accessed. *
If your study has not produced novel datasets, please mention this fact in the Data Availability Section.

8) We would also encourage you to include the source data for figure panels that show essential data. Numerical data should be provided as individual .xls or .csv files (including a tab describing the data). For blots or microscopy, uncropped images should be submitted (using a zip archive if multiple images need to be supplied for one panel). Additional information on source data and instruction on how to label the files are available at
<https://www.embopress.org/page/journal/14693178/authorguide#sourcedata>.

9) Our journal also encourages inclusion of *data citations in the reference list* to directly cite datasets that were re-used and obtained from public databases. Data citations in the article text are distinct from normal bibliographical citations and should directly link to the database records from which the data can be accessed. In the main text, data citations are formatted as follows: "Data ref: Smith et al, 2001" or "Data ref: NCBI Sequence Read Archive PRJNA342805, 2017". In the Reference list, data citations must be labeled with "[DATASET]". A data reference must provide the database name, accession number/identifiers and a resolvable link to the landing page from which the data can be accessed at the end of the reference. Further instructions are available at https://www.embopress.org/page/journal/14693178/authorguide#referencesformat

10) Regarding data quantification (see Figure Legends:
https://www.embopress.org/page/journal/14693178/authorguide#figureformat)

The following points must be specified in each figure legend:

- the name of the statistical test used to generate error bars and P values,

- the number (n) of independent experiments (please specify technical or biological replicates) underlying each data point,

- the nature of the bars and error bars (s.d., s.e.m.),

- If the data are obtained from n {less than or equal to} 2, use scatter blots showing the individual data points.

Discussion of statistical methodology can be reported in the materials and methods section, but figure legends should contain a basic description of n, P and the test applied.

- Please also include scale bars in all microscopy images.

11) The journal requires a statement specifying whether or not authors have competing interests (defined as all potential or actual interests that could be perceived to influence the presentation or interpretation of an article). In case of competing interests, this must be specified in your disclosure statement. Further information: https://www.embopress.org/competing-interests

We would also welcome the submission of cover suggestions, or motifs to be used by our Graphics Illustrator in designing a cover.

As part of the EMBO publication's Transparent Editorial Process, EMBO reports publishes online a Review Process File (RPF) to accompany accepted manuscripts. This File will be published in conjunction with your paper and will include the referee reports, your point-by-point response and all pertinent correspondence relating to the manuscript.

You are able to opt out of this by letting the editorial office know (emboreports@embo.org). If you do opt out, the Review Process File link will point to the following statement: "No Review Process File is available with this article, as the authors have chosen not to make the review process public in this case."

I look forward to seeing a revised form of your manuscript when it is ready. Please use this link to submit your revision: https://embor.msubmit.net/cgi-bin/main.plex

Best,
Esther

Esther Schnapp, PhD
Senior Editor
EMBO reports

Referee #1:

Radecke et al. present a study on morphological correlates of synaptic vesicle (SV) exocytosis entitled "Morphofunctional changes at the active zone during synaptic vesicle exocytosis". This study sheds light on transient stages of exocytosis, which SVs need to undergo prior to a full collapse into the membrane and on SV tethering during activity changes. To be able to visualize short-living intermediates in correlation to function, a high-resolution technique that preserves activity changes in a millisecond range is required.
Radecke et al. chose one of the most challenging combinations, cryo-electron tomography (ET) of synapses combined with a direct stimulation prior to plunge-freezing. Different activity states were obtained by a specific spray-technique of a high potassium solution for depolarization onto grids with rat synaptosomal preparations. Additionally, the authors took advantage of two mutant mouse lines with mutations in the SNARE-complex protein SNAP-25 that arrest or disinhibit spontaneous release (freezing cell cultures). These two approaches required some specific methods to be able to perform cryo-ET, (i) the authors used correlative light and electron microscopy to identify synaptosomes that were stimulated by the high-potassium solution, which additionally contained fluorescein. Moreover, (ii) they made use of a technique to grow neuronal cultures on grids in an efficient way, but still remaining thin enough to be suitable for plunge-freezing and cryo-ET. Therefore, the full method flow is certainly of interest for a broader neuroscience community.
The authors than analyzed morphological parameters such as SV membrane and plasma membrane curvature as well as the number and the tethering state of SVs. As widely done in morphological studies of synapses, the authors quantified the abundance of membrane-proximal, intermediate and further distal SVs together with the number of tethers to other SVs or to the AZ membrane. It needs to be mentioned that only cryo-ET can resolve the tethering state for each SV in a native, hydrated state and therefore is the best method to investigate if and how tethering changes during altered activity.

The key findings of this study are:

Upon stimulation, early fusion events are accompanied by a buckling of the active zone membrane and the SV membrane. After this buckling, the fusion pore formed and finally a full collapse could be observed. It needs to be mentioned that these stages are not precisely temporally resolved. Instead, the authors arranged the observed stages at depolarized AZs in the most logical order. Moreover, upon depolarization, the authors found that proximal tethered SVs formed additional tethers to the PM and connections between SVs reduced. Both SNAP-25 mutants (inactive and over-active) revealed a loss of connectors, while the disinhibiting mutation also caused a loss of membrane proximal multiple-tethered SVs.
The presented data largely confirm previous findings of other cryo-ET and high-pressure freezing/freeze substitution/ET studies. However, the authors present more details here and observed a deformation of SVs towards the plasma membrane together with the buckling of the plasma membrane prior to SV fusion. Moreover, they saw a loss of connected vesicles in membrane-proximity, which might allow the vesicles to be recruited to the AZ.
Overall, the study is carefully done, the figures are clear and the manuscript contains a comprehensive quantification of SV pools under different activity states using a challenging experimental approach. As the authors state in the manuscript, the spray method is not temporally precise, because the exact delay between stimulation and freezing cannot be determined for each tomogram individually. Still, I agree with the authors that this might be one of the closest coupling so far achieved for a cryo-EM technique.
I have a few points that are not fully clear to me:
I am struggling a bit with the exocytosis intermediates. As previously published by Imig et al., 2014, who investigated hippocampal synapses after high-pressure freezing, freeze substitution and electron tomography, the order of exocytosis steps presented in this manuscript seems very likely, maybe this study should be mentioned in this context as well. Already Imig et al. described the buckling of the AZ membrane towards a docked SV but could not observe the bending of the SV membrane. Moreover, less pronounced membrane curvature events might have been missed before. Likely, such stages can only be visualized using cryo-techniques.
-The author clearly say that they did not observe these intermediate events at non-sprayed AZs. Due to technical limitations they did not image within a region on the grid, which was covered by the solution, they only chose synaptosomes that revealed an accessible AZ but were partially overlayed by a high-potassium solution droplet and assumed that via diffusion a stimulation to an unknown time point within the calculated range took place. My question is, could the authors observe in every as "sprayed and stimulated" synaptosome/AZ an exocytosis intermediate event? Or maybe more than one? Or were there also

synaptosomes that did not show any signs of exocytosis?
The authors do not mention any numbers of the observed exocytosis events, I assume they are still rare, even after depolarization. However, these events are the basis of sorting the synapses in late and early exocytosis stages and the whole quantification is relying on this categorization. Therefore, a clear description of the frequency of these events is mandatory. How many late events are observed, how many early events and what is the exact definition late vs. early? Here, a scheme might help.
In summary, the presented study gives insight into transient SV stages and SV tethering during exocytosis with highest resolution and structural preservation. The content of the MS is only partially new but the used method flow is very advanced and challenging and the combination with two SNAP-25 mutants gives a more complete view on morphological events that take place during SV exocytosis. Therefore, this study is certainly of interest for the neuroscience community. In principle, I favor publication in EMBO Reports, but the mentioned issues need to be clarified.

Referee #2:

I have reviewed this manuscript for a previous submission, and the authors have responded to my different concerns. Here I review their replies and the revised manuscript.
Overall, the authors have made serious attempts to resolve the problems I indicated, but it seems like these issues cannot be fully answered.
My main point was that the authors use "signs of exocytosis" to identify the temporal phase in which each synapse finds itself, and then analyze the same "signs of exocytosis" to determine what happens in each temporal phase. This constitutes circular reasoning, and is not a correct scientific procedure.
The authors' reply to this comment was that "the order that we suggest relies on information obtained through other methods. Indeed, several studies have shown that after Ca2+ influx, the coordinated action of synaptotagmin-1 and the SNARE complex first buckles the plasma membrane and then the two lipid membranes enter in contact and engage in hemi-fusion and fusion. Any other order than the proposed one would not make sense in a process occurring in about 100 µs. We could not envisage an omega figure to occur before the bending of the plasma membrane. Again, the uniqueness of our study lies in the fact that it brings for the first time a direct observation of the very early stages of synaptic vesicle exocytosis". In other words, the authors KNOW what they expect to see, based on other publications and other methods, and therefore they KNOW what the results should be. This enables them to then TEST what the results actually are. This is circular reasoning, and remains an incorrect scientific procedure.
To place this into perspective, one can perceive several different stages of exocytosis within a single synapse frozen at 2.5 ms after stimulation, in the work of Torri-Tarelli et al., J Cell Biol, 1985 (doi: 10.1083/jcb.101.4.1386.). This is probably due to a fairly long time span during which freezing takes place, of several milliseconds (as noted in the respective paper), during which some vesicles have the time to fuse fully, while others do not. What would be the exocytosis stage of that synapse, then? Should we say that the respective single synapse (and single EM section) represents several time points after stimulation, one for each synaptic vesicle found in a different morphological state? This would be absurd to suggest, and thus indicates how dangerous it is to use the morphological characteristics of the sample to infer the stimulation time.
The authors still show no statistics to indicate that they can only see the exocytosis signs in stimulated synapses. They mention that they observed no such signs in "hundreds of images". Were these images analyzed in a blind fashion? If this was not the case, than such an analysis is not relevant.
When I questioned the novelty of the work, the authors replied that "Uel McMahan's pioneering work has been performed on frog neuromuscular junction. There, tethers and docked synaptic vesicles are ordered in highly regular rows at the active zone plasma membrane. This is very different to the situation in central nervous system synapses, which we report." They should be aware of work of the McMahan laboratory performed at the mouse neuromuscular junction (Nagwaney et al., J Comp Neurol, 2009, DOI: 10.1002/cne.21975), which shows connectors more similar to those in the rodent CNS. Overall, the work presented here still has very limited novelty, as long as it is based on circular reasoning.
For the statistics, the authors note that they now use a Benjamini-Hochberg correction procedure for multiple testing. However, they fail to report the false discovery rate (FDR) of the procedure, which makes this correction unclear.
The authors concede that they use a hyper-osmotic solution to stimulate the grids. This is not optimal, irrespective of the comments of the authors.
Overall, I am still unable to suggest that the manuscript be published.

Cross-comments from referee 1:

I think the comments of the other referee are correct, however, I was judging it a bit milder. We raised the same point regarding the statistics of the exocytosis events, even if mentioned in the point to point response, the authors did not clearly define their late and early stage signs of exocytosis and did not mention numbers specifically in the MS. Such statements/statistics need to be included in the MS, not only in the rebuttal letter (I think I forgot to clearly state this in my review). Moreover, it needs to be secured, that an assumed late stage is really late stage of exocytosis, pure assumption is not enough. That is the weak point, they cannot correlate late and early signs of exocytosis to any accurate time point, the order of these events is, even if a likely

one, as said, only an assumption. They toned down their statement about temporal precision in the revised version, but the problem remains. If they cannot provide a convincing way to define late and early stages, the whole interpretation could be wrong. Statistics and a clear way of defining late and early is required, the best would be a defined time point.

Furthermore, yes, the results are really only to a small extent new, I also raised this point. But when proper presented and validated, I find the method combination interesting and worth to be published. But, as said, only if the late_early stage sorting has a substantial basis.

Advisor's comments:

Radecke, Seeger et al in this study use cryo-ET to study the ultrastructural changes that occur during stimulated neuroexocytosis. The authors use two models, rat brain synaptosomes and cultured mouse hippocampal neurons grown on EM grids placed on top of an astrocyte layer, paired with spraying a depolarizing buffer solution and rapid plunge freezing in liquid ethane. A key feature of the approach developed is that due to the stochastic distribution of the buffer droplets synaptic vesicles (SVs) may either have fused with a delay (of about 7-35 ms as the authors estimate) or within the last ms or so depending on their distance to the sprayed droplet. The results and the authors' interpretations of these can be summarized as follows: (1) Chronological ordering of the obtained snapshots according to common sense and prior data in various systems suggests that the onset of SV fusion is associated with plasma membrane buckling, possibly as a result of SNARE-zippering or Synaptotagmin penetration into membranes. (2) Stimulation results in the depletion of docked SVs within a zone of 0-50 nm from the AZ. This is further confirmed in synapses from SNAP-25 KO mice rescued by re-expression of SNAP-25 mutants with decreased (4E) or increased (4K) fusion activity. Finally (3), it is shown that chemical stimulation increases tether formation on membrane-proximal vesicles and induces a slower transient rise in the number of intervesicular connections between SVs. Abnormal exocytic activity in the SNAP-25 4E or 4K mutants also affects intervesicular connectivity in the distal region, e.g. the recycling SV pool.

I understand that this is a revised manuscript version that has been resubmitted following an initial round of review. The authors report on the technically very challenging analysis of SV redistribution, tethering and connectivity in two relevant models of neuroexocytosis. Overall, the experiments are generally well done and the results should be of interest to the readers of EMBO Reports. The presentation of the data and writing of the manuscript should be improved, however. In addition, I have a few minor points that ought to be addressed.

1. At least in the version supplied to me figures 3-5 appeared somewhat blurry, contained multiple font types and sizes, and would benefit from a revised layout. The message derived from the SV distribution curves shown in Figs. 5A,B needs to be distilled out better, perhaps by adding high mag insets for proximal SVs and/ or the addition of bar graphs illustrating the key conclusions. Similar concerns apply to Figs. 3A,B.
2. Another example for lack of clarity when it comes to figures vs. text is Fig. 5C. The graph in the figure is a bar plot representing the fraction of connected SVs as a function of distance. The text refers to significant differences between distal 2 region and the proximal region. However, none of these are marked in the actual figure. This is confusing and will annoy non-expert readers.
3. Figure legends would need to make clear what n represents, e.g. in Figs. 3C,D, 4, 5E-H. Are these independent experiments, data from individual synaptosomes? This should then also be explicitely said in the data analysis and statistics section of the methods.
4. Figure 3D is not quoted in the text. Are the differences between non-sprayed, early and late fusion statistically significant?
5. In the 4K mutant the RRP is greatly depleted according to the morphological criteria defined by the authors. Has this been verified electrophysiologically before? If so, the corresponding reference should be quoted.
6. Some figures (e.g. Figure 5B) would be more convincing, if additional examples of EM tomograms were shown.
7. Fig. S3 should contain segmented tomograms of WT, 4E and 4K synapses, not just 4E.
8. It might be beneficial to include the scheme (or parts of it) in Fig S1 into Fig 1.
9. Discussion: As there are a number of uncertainties related to the technique (e.g. the exact time point of fusion, order of events, identity of tethers and connectors) it would be useful to include a short paragraph on the limitations of the study and the approach and how these could be overcome.

More cross-comments from referee 1:

The problem with the study is that the authors more or less assume late and early stages of exocytosis according to a certain morphology of SVs. (i) These events are rare and they did not provide a statistic on that, (ii) they do not clearly define criteria (morphological) for late and early stages of exocytosis and (iii) they sorted and analysed their data according to this assumption. If their assumption of the order/interpretation late/early exocytosis is not correct, the data are also not analysed correctly. They need to be clear with the statistics, how often is which feature occuring at the AZs, the number of tomograms is not so high (difficult method, this I fully understand and I highly appreciate the method flow). Therefore, for each tomogram they should

name and also show in a supplement figure the morphological late/early staged SVs. They need to convince the reader that this categorization is correct.

Answers to Refees' comments

Referee #1:
Radecke et al. present a study on morphological correlates of synaptic vesicle (SV) exocytosis entitled "Morphofunctional changes at the active zone during synaptic vesicle exocytosis". This study sheds light on transient stages of exocytosis, which SVs need to undergo prior to a full collapse into the membrane and on SV tethering during activity changes. To be able to visualize short-living intermediates in correlation to function, a high-resolution technique that preserves activity changes in a millisecond range is required.
Radecke et al. chose one of the most challenging combinations, cryo-electron tomography (ET) of synapses combined with a direct stimulation prior to plunge-freezing. Different activity states were obtained by a specific spray-technique of a high potassium solution for depolarization onto grids with rat synaptosomal preparations. Additionally, the authors took advantage of two mutant mouse lines with mutations in the SNARE-complex protein SNAP-25 that arrest or disinhibit spontaneous release (freezing cell cultures). These two approaches required some specific methods to be able to perform cryo-ET, (i) the authors used correlative light and electron microscopy to identify synaptosomes that were stimulated by the high-potassium solution, which additionally contained fluorescein. Moreover, (ii) they made use of a technique to grow neuronal cultures on grids in an efficient way, but still remaining thin enough to be suitable for plunge- freezing and cryo-ET. Therefore, the full method flow is certainly of interest for a broader neuroscience community.
The authors than analyzed morphological parameters such as SV membrane and plasma membrane curvature as well as the number and the tethering state of SVs. As widely done in morphological studies of synapses, the authors quantified the abundance of membrane-proximal, intermediate and further distal SVs together with the number of tethers to other SVs or to the AZ membrane. It needs to be mentioned that only cryo-ET can resolve the tethering state for each SV in a native, hydrated state and therefore is the best method to investigate if and how tethering changes during altered activity.
The key findings of this study are:
Upon stimulation, early fusion events are accompanied by a buckling of the active zone membrane and the SV membrane. After this buckling, the fusion pore formed and finally a full collapse could be observed. It needs to be mentioned that these stages are not precisely temporally resolved. Instead, the authors arranged the observed stages at depolarized AZs in the most logical order. Moreover, upon depolarization, the authors found that proximal tethered SVs formed additional tethers to the PM and connections between SVs reduced. Both SNAP-25 mutants (inactive and over-active) revealed a loss of connectors, while the disinhibiting mutation also caused a loss of membrane proximal multiple-tethered SVs.
The presented data largely confirm previous findings of other cryo-ET and high-pressure freezing/freeze substitution/ET studies. However, the authors present more details here and observed a deformation of SVs towards the plasma membrane together with the buckling of the plasma membrane prior to SV fusion. Moreover, they saw a loss of connected vesicles in membrane-proximity, which might allow the vesicles to be recruited to the AZ.
Overall, the study is carefully done, the figures are clear and the manuscript contains a comprehensive quantification of SV pools under different activity states using a challenging experimental approach. As the authors state in the manuscript, the spray method is not

temporally precise, because the exact delay between stimulation and freezing cannot be determined for each tomogram individually. Still, I agree with the authors that this might be one of the closest coupling so far achieved for a cryo-EM technique.

We are thankful to the referee for his appreciation of our work.

I have a few points that are not fully clear to me:
I am struggling a bit with the exocytosis intermediates. As previously published by Imig et al., 2014, who investigated hippocampal synapses after high-pressure freezing, freeze substitution and electron tomography, the order of exocytosis steps presented in this manuscript seems very likely, maybe this study should be mentioned in this context as well. Already Imig et al. described the buckling of the AZ membrane towards a docked SV but could not observe the bending of the SV membrane. Moreover, less pronounced membrane curvature events might have been missed before. Likely, such stages can only be visualized using cryo-techniques.

We thank the reviewer for the advice. We have now commented the similarities and differences of our results with respect to the ones of Imig et al.

-The author clearly say that they did not observe these intermediate events at non-sprayed AZs. Due to technical limitations they did not image within a region on the grid, which was covered by the solution, they only chose synaptosomes that revealed an accessible AZ but were partially overlayed by a high-potassium solution droplet and assumed that via diffusion a stimulation to an unknown time point within the calculated range took place. My question is, could the authors observe in every as "sprayed and stimulated" synaptosome/AZ an exocytosis intermediate event? Or maybe more than one? Or were there also synaptosomes that did not show any signs of exocytosis?

We did not always detect signs of exocytosis in sprayed synaptosomes. This was expected because the release probability of a synapse under depolarization is significantly lower than 1. For example, Slutsky et al. 2004 (10.1016/j.neuron.2004.11.013) and Branco et al. 2008 10.1016/j.neuron.2008.07.006 reported a release probability distribution in hippocampal synapses, which was strongly skewed towards low values, with a median between 0.14 and 0.22. Furthermore, the limited spatial resolution of cryo-fluorescence microscopy may have also contributed to this. We occasionally observed more than one event in a single synapse. Yet we did not observe simultaneously early and late events in a single synapse.
To be clear, when we mention non-sprayed synaptosomes, we mean that the EM grid was not sprayed at all

The authors do not mention any numbers of the observed exocytosis events, I assume they are still rare, even after depolarization. However, these events are the basis of sorting the synapses in late and early exocytosis stages and the whole quantification is relying on this categorization. Therefore, a clear description of the frequency of these events is mandatory.

How many late events are observed, how many early events and what is the exact definition late vs. early? Here, a scheme might help.

We have observed the following number of exocytosis events (referring to figure 2 lettering scheme): B, 8; C, 3; D, 3; E, 2; F, 3; G, 1; H, 11. Events of type B to E were classified as early, while events of type F to H were classified as late. We have added this piece of information in the figure caption and in the results.

In summary, the presented study gives insight into transient SV stages and SV tethering during exocytosis with highest resolution and structural preservation. The content of the MS is only partially new but the used method flow is very advanced and challenging and the combination with two SNAP-25 mutants gives a more complete view on morphological events that take place during SV exocytosis. Therefore, this study is certainly of interest for the neuroscience community. In principle, I favor publication in EMBO Reports, but the mentioned issues need to be clarified.

Referee #2:
I have reviewed this manuscript for a previous submission, and the authors have responded to my different concerns. Here I review their replies and the revised manuscript.
Overall, the authors have made serious attempts to resolve the problems I indicated, but it seems like these issues cannot be fully answered. My main point was that the authors use "signs of exocytosis" to identify the temporal phase in which each synapse finds itself, and then analyze the same "signs of exocytosis" to determine what happens in each temporal phase. This constitutes circular reasoning, and is not a correct scientific procedure.
The authors' reply to this comment was that "the order that we suggest relies on information obtained through other methods. Indeed, several studies have shown that after Ca^{2+} influx, the coordinated action of synaptotagmin-1 and the SNARE complex first buckles the plasma membrane and then the two lipid membranes enter in contact and engage in hemi-fusion and fusion. Any other order than the proposed one would not make sense in a process occurring in about 100 µs. We could not envisage an omega figure to occur before the bending of the plasma membrane. Again, the uniqueness of our study lies in the fact that it brings for the first time a direct observation of the very early stages of synaptic vesicle exocytosis". In other words, the authors KNOW what they expect to see, based on other publications and other methods, and therefore they KNOW what the results should be. This enables them to then TEST what the results actually are. This is circular reasoning, and remains an incorrect scientific procedure.

We agree with the reviewer that the results we present here are complex and require careful reasoning.

As we now state in the manuscript, we use bent lipid membranes as signs of exocytosis because these were found only on sprayed grids. Among those events, we distinguished the late fusion state from the early one by opened SV fusion pore, as opposed to a point contact between an SV and the plasma membrane or bent membranes without a lipid contact. The open SV pores were described before, albeit only in EM preparations based on dehydrating and staining, while early fusion events were only speculated but not observed in synapses before. The temporal ordering of the early and the late phase is dictated by the current

understanding of neurotransmitter release, and it was called “common sense” by the advisor. Therefore, the first major result of our study is that we observed and described different early fusion events for the first time, and that we observed he late fusion event (open fusion pore) without EM preparation artefacts.

As the reviewer correctly stated, we used signs of exocytosis to identify the temporal state of the synapse (early or late fusion stage). However, while these signs were based solely on the lipid curvature, we proceeded to analyze SV-bound protein complexes (tethers and connectors) and the precise localization of all SVs, completely different entities. Therefore, we did not “analyze the same signs of exocytosis”, hence we did not employ circular reasoning.

Related, the quantitative characterization of tethers and connectors in non-stimulated, early and late fusion stages constitutes the second major result of our study.

To answer the last part of this point raised by the reviewer, yes we expected to see open SV fusion pores, so we confirmed previous observations using a better method. As the early fusion events were only speculated it cannot be stated that we expected them. We simply observed events that were caused by synaptic stimulation and had to occur before the open SV fusion pores are formed. Furthermore, we did not know what the tethers and connectors results would be, so we did not “test what the results actually are”. Therefore, there is no circular reasoning in our presentation.

To place this into perspective, one can perceive several different stages of exocytosis within a single synapse frozen at 2.5 ms after stimulation, in the work of Torri-Tarelli et al., J Cell Biol, 1985 (doi: 10.1083/jcb.101.4.1386.). This is probably due to a fairly long time span during which freezing takes place, of several milliseconds (as noted in the respective paper), during which some vesicles have the time to fuse fully, while others do not. What would be the exocytosis stage of that synapse, then? Should we say that the respective single synapse (and single EM section) represents several time points after stimulation, one for each synaptic vesicle found in a different morphological state? This would be absurd to suggest, and thus indicates how dangerous it is to use the morphological characteristics of the sample to infer the stimulation time.

The referee writes that Torri-Tarelli et al observed multiple stages of exocytosis in a single synapse. It is important to consider that Torri-Tarelli et al pre-treated their samples with 1mM 4-aminopyridine (4-AP). 4-AP is a blocker of voltage-gated potassium channels and is used to enhance dramatically exocytotic activity upon electrical stimulation. Heuser et al introduced its use in their landmark paper (JCB 1979; 10.1083/jcb.81.2.275). Below, we reproduce Figure 3 of the Heuser et al paper, which shows a 50-fold increase in transmitter release due to 4-AP as measured by electrophysiology.

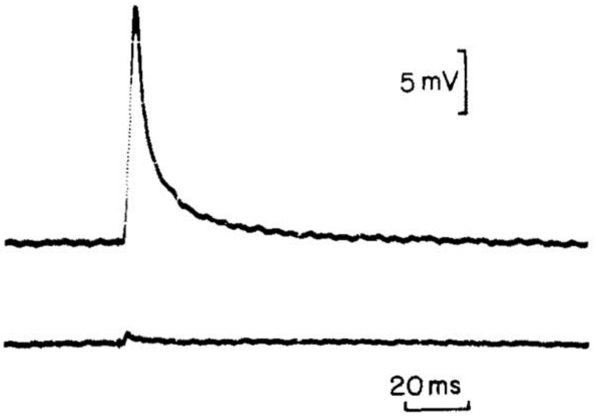

FIGURE 3 Intracellular EPPs recorded from a single muscle fiber in 10^{-4} M curare and 10 mM Ca^{++}, before (below) and after (above) exposure to 1 mM 4-AP. The increase of transmitter output produced by 4-AP is about 50-fold.

Table 1 of the same paper is reproduced below and shows that 1mM 4-AP leads to a 50-fold increase in the number of vesicles trapped in exocytosis and seen by EM (column 3). Moreover, and most importantly column 5 shows that while without 4-AP 99% of the vesicles trapped in exocytosis have already collapsed, this number decreases to 23% in presence of 1mM 4-AP. The remaining 77% are open but not yet collapsed. This clearly demonstrates that the use of 4-AP disrupts exocytosis synchronicity.

TABLE I

Synaptic Vesicle Openings vs. Quantal Content

(1) Concn of 4-AP (in 10 mM Ca^{++})	(2) Average quantal content of EPP	(3) Open and collapsed vesicles, per average endplate	(4) Just collapsed vesicles	(5) % Collapsed vesicles	(6) No. of muscles	(7) No. of active zones
M						
0	180	130	126	99+	4	75
10^{-5}	700	800	320	40	4	77
10^{-4}	1,900	2,100	770	37	9	262
10^{-3}	4,600	5,300	1,200	23	2	26

Comparison between the number of quanta discharged (estimated by the curare dose-ratio method in Appendix 2) vs. the number of vesicles caught in the act of exocytosis (both those that are wide open and those that have collapsed) in three concentrations of 4-AP. Column 2 should be compared with column 3 to see how good the correlation is. Column 5 illustrates that it becomes progressively harder to catch vesicles in the wide-open state as 4-AP concentration is reduced and the total number of vesicle openings declines.

Consistently, in our study, where no 4-AP was used, we have not observed simultaneous occurrence of early and late events in any single synapse. Therefore, the referee's concern is not relevant.

The authors still show no statistics to indicate that they can only see the exocytosis signs in stimulated synapses. They mention that they observed no such signs in "hundreds of images". Were these images analyzed in a blind fashion? If this was not the case, than such an analysis is not relevant.

We have now added the number of events of each type of exocytosis event in our manuscript (see our answer to referee #1). We also state in the manuscript that we did not observe such structures in synapses that have not been sprayed with high KCl. These events are obvious to detect (see Figure 2), hence there is no need for blind analysis.

When I questioned the novelty of the work, the authors replied that "Uel McMahan's pioneering work has been performed on frog neuromuscular junction. There, tethers and docked synaptic vesicles are ordered in highly regular rows at the active zone plasma membrane. This is very different to the situation in central nervous system synapses, which we report." They should be aware of work of the McMahan laboratory performed at the mouse neuromuscular junction (Nagwaney et al., J Comp Neurol, 2009, DOI: 10.1002/cne.21975), which shows connectors more similar to those in the rodent CNS. Overall, the work presented here still has very limited novelty, as long as it is based on circular reasoning.

Cryo-ET is based on a sample preparation that preserves the native state of the specimen and that allows direct imaging of proteins and lipids at a single nanometer scale. Therefore, it allows interpretation at the molecular level. This is in contrast with the preparation used in Nagwaney et al., J Comp Neurol, 2009, which employed chemical fixation, dehydration and staining. While these procedures were used to reach some seminal discoveries in cell biology, they are known to induce rearrangements of proteins and lipids.

In one of the previous answers, we stated two major results of this study and showed that we did not employ circular reasoning. The third major result that the reviewer apparently did not consider is the analysis of SV-bound protein complexes in SNAP25 mutants that cause significant and opposing effects on SV fusion.

For the statistics, the authors note that they now use a Benjamini-Hochberg correction procedure for multiple testing. However, they fail to report the false discovery rate (FDR) of the procedure, which makes this correction unclear.

We have used a python implementation of the Benjamini-Hochberg correction in which the FDR does not need to be input. Instead the function returns corrected P-values. This variation of the original Benjamini-Hochberg correction algorithm was proposed by Yekutieli and Benjamini (doi 10.1016/s0378-3758(99)00041-5)].
If a corrected P-value is smaller than the defined acceptable false discovery rate, then the null hypothesis is rejected, i.e. the difference is considered statistically significant.
This algorithm enables to test multiple false discovery rates in one step and its conclusions are exactly the same as the original Benjamini-Hochberg correction algorithm run multiple times with different false discovery rates. In the figures, ***, **, and * indicate a corrected P-value lower than false discovery rates of 0.001, 0.01, and 0.05, respectively. We have added details in the materials and methods.

Cross-comments from referee 1:
I think the comments of the other referee are correct, however, I was judging it a bit milder. We raised the same point regarding the statistics of the exocytosis events, even if mentioned in the point to point response, the authors did not clearly define their late and early stage signs of exocytosis and did not mention numbers specifically in the MS. Such statements/statistics need to be included in the MS, not only in the rebuttal letter (I think I forgot to clearly state this in my review).

We added a clear criterion for the early and the late fusion events to the manuscript. We answered this in more detail above. In short, bent membranes are a sign of a fusion event. Late fusion events are characterized by an open SV fusion pore, while early fusion show no or a point contact between an SV and the plasma membrane. We have now also added the requested numbers in the manuscript. Please note that the numbers are higher than in those in the previous rebuttal letter because we went through an additional set of tomograms that we omitted not include previously.

Moreover, it needs to be secured, that an assumed late stage is really late stage of exocytosis, pure assumption is not enough. That is the weak point, they cannot correlate late and early signs of exocytosis to any accurate time point, the order of these events is, even if a likely one, as said, only an assumption. They toned down their statement about temporal precision in the revised version, but the problem remains. If they cannot provide a convincing way to define late and early stages, the whole interpretation could be wrong. Statistics and a clear way of defining late and early is required, the best would be a defined time point.

Furthermore, yes, the results are really only to a small extent new, I also raised this point. But when proper presented and validated, I find the method combination interesting and worth to be published. But, as said, only if the late_early stage sorting has a substantial basis.

It is firmly established in the field that an open SV fusion pore characterizes the last stage of full-collapse exocytosis. Furthermore, it would be hard to imagine that bent membranes and point contact between an SV and the plasma membrane happens after the exocytosis. Therefore, the temporal ordering of the fusion stages is unambiguous, the advisor described the ordering “common sense”.

As mentioned above, we also clearly state the number of events and explain the statistics used.

We thank the referee for the appreciation of our methods and results.

Advisor's comments:
Radecke, Seeger et al in this study use cryo-ET to study the ultrastructural changes that occur during stimulated neuroexocytosis. The authors use two models, rat brain

synaptosomes and cultured mouse hippocampal neurons grown on EM grids placed on top of an astrocyte layer, paired with spraying a depolarizing buffer solution and rapid plunge freezing in liquid ethane. A key feature of the approach developed is that due to the stochastic distribution of the buffer droplets synaptic vesicles (SVs) may either have fused with a delay (of about 7-35 ms as the authors estimate) or within the last ms or so depending on their distance to the sprayed droplet. The results and the authors' interpretations of these can be summarized as follows: (1) Chronological ordering of the obtained snapshots according to common sense and prior data in various systems suggests that the onset of SV fusion is associated with plasma membrane buckling, possibly as a result of SNARE-zippering or Synaptotagmin penetration into membranes. (2) Stimulation results in the depletion of docked SVs within a zone of 0-50 nm from the AZ. This is further confirmed in synapses from SNAP-25 KO mice rescued by re-expression of SNAP-25 mutants with decreased (4E) or increased (4K) fusion activity. Finally (3), it is shown that chemical stimulation increases tether formation on membrane-proximal vesicles and induces a slower transient rise in the number of intervesicular connections between SVs. Abnormal exocytic activity in the SNAP-25 4E or 4K mutants also affects intervesicular connectivity in the distal region, e.g. the recycling SV pool.
I understand that this is a revised manuscript version that has been resubmitted following an initial round of review. The authors report on the technically very challenging analysis of SV redistribution, tethering and connectivity in two relevant models of neuroexocytosis. Overall, the experiments are generally well done and the results should be of interest to the readers of EMBO Reports. The presentation of the data and writing of the manuscript should be improved, however. In addition, I have a few minor points that ought to be addressed.

We are grateful to the adviser for their appreciation of our work and for the suggestions for improvement.

1. At least in the version supplied to me figures 3-5 appeared somewhat blurry, contained multiple font types and sizes, and would benefit from a revised layout. The message derived from the SV distribution curves shown in Figs. 5A,B needs to be distilled out better, perhaps by adding high mag insets for proximal SVs and/ or the addition of bar graphs illustrating the key conclusions. Similar concerns apply to Figs. 3A,B.

We are sorry about the blurry quality, which might have resulted from compression due to file size limit imposed by the submission system.

2. Another example for lack of clarity when it comes to figures vs. text is Fig. 5C. The graph in the figure is a bar plot representing the fraction of connected SVs as a function of distance. The text refers to significant differences between distal 2 region and the proximal region. However, none of these are marked in the actual figure. This is confusing and will annoy non-expert readers.

Thank you for the pointing out this confusing point. We have added the name of the distance groups in the figures.

3. Figure legends would need to make clear what n represents, e.g. in Figs. 3C,D, 4, 5E-H. Are these independent experiments, data from individual synaptosomes? This should then also be explicitly said in the data analysis and statistics section of the methods.

For every plot we have now included the n's and what they represent directly in the figures. We have also improved the description of the statistics used in the figure captions.

4. Figure 3D is not quoted in the text. Are the differences between non-sprayed, early and late fusion statistically significant?

Indeed, there was no significant difference and we have now included a mention of it in the results.

5. In the 4K mutant the RRP is greatly depleted according to the morphological criteria defined by the authors. Has this been verified electrophysiologically before? If so, the corresponding reference should be quoted.

Yes we have shown this previously (Ruiter et al 2019. 10.1016/j.celrep.2019.01.103). We had mentioned it in the previous version of the manuscript but we have made it clearer now.

6. Some figures (e.g. Figure 5B) would be more convincing, if additional examples of EM tomograms were shown.
7. Fig. S3 should contain segmented tomograms of WT, 4E and 4K synapses, not just 4E.

We have addressed these two points (The figure is now numbered S2).
8. It might be beneficial to include the scheme (or parts of it) in Fig S1 into Fig 1.

We have moved it to the main (it is now Fig. 2).

9. Discussion: As there are a number of uncertainties related to the technique (e.g. the exact time point of fusion, order of events, identity of tethers and connectors) it would be useful to include a short paragraph on the limitations of the study and the approach and how these could be overcome.

We have added a paragraph about the uncertainties and limitations of the study in the discussion.

More cross-comments from referee 1:
The problem with the study is that the authors more or less assume late and early stages of exocytosis according to a certain morphology of SVs. (i) These events are rare and they did not provide a statistic on that, (ii) they do not clearly define criteria (morphological) for late and early stages of exocytosis and (iii) they sorted and analysed their data according to this assumption. If their assumption of the order/interpretation late/early exocytosis is not correct, the data are also not analysed correctly. They need to be clear with the statistics, how often is which feature occuring at the AZs, the number of tomograms is not so high (difficult method, this I fully understand and I highly appreciate the method flow). Therefore, for each tomogram they should name and also show in a supplement figure the

morphological late/early staged SVs. They need to convince the reader that this categorization is correct.

All these points have been addressed and commented above.

Dear Dr. Zuber,

Thank you for the submission of your revised manuscript. We have now received the enclosed report from referee 1 who was asked to assess it. Referee 1 still has a few more suggestions that I would like you to address and incorporate before we can proceed with the official acceptance of your manuscript. Please co-submit a point-by-point response to these last comments.

A few editorial requests will also need to be addressed:

- In the author checklist the questions on statistics should be answered. You can also just enter a note in the checklist on where in the ms this information can be found.

- Please add up to 5 keywords to the ms file.

- The REFERENCE FORMAT needs to be corrected to the named EMBO reports (Harvard) style.

- Funding information should only be included in the Acknowledgement section

- For figures Fig 2A, EV2A+B, EV3C-F, EV4A+B callouts are missing, please add.

- Figs 3,5,6 panel callouts are not alphabetically ordered, please correct.

- The movies and their legends should be ZIPd together and uploaded as one file per movie. The movie legends need to be removed from the manuscript file.

- The EV tables should be uploaded individually using the file type Expanded View (Expanded View Table 1, etc).

- The figure labeling needs correcting, i.e. Fig 1A1 should be Figure 1Ai.

- I attach to this email a related ms file with comments by our data editors. Please address all comments in the final ms.

EMBO press papers are accompanied online by A) a short (1-2 sentences) summary of the findings and their significance, B) 2-3 bullet points highlighting key results and C) a synopsis image that is exactly 550 pixels wide and 200-600 pixels high (the height is variable). You can either show a model or key data in the synopsis image. Please note that text needs to be readable at the final size. Please send us this information along with the revised manuscript.

Regarding the abstract, please double-check that it contains no overstatements. It currently says "time-resolved" and "early" and "late" fusion. If I understand the referees correctly, this must be corrected.

I look forward to seeing a final version of your manuscript as soon as possible. Please use this link to submit your revision:
https://embor.msubmit.net/cgi-bin/main.plex

Best regards,
Esther

Esther Schnapp, PhD
Senior Editor
EMBO reports

Referee #1:

Radecke et al. significantly improved the quality of the manuscript. I highly appreciate that also new tomograms were acquired and included in the analysis.
Even though the exocytosis events remain rare, which might simply have biological reasons (could be discussed by the authors), the authors now better worked out the basis of sorting stimulated synapses in "late" and "early" staged exocytosis events.

However, some points remain to be clarified before publication:
In the end, the authors have a temporal range of stimulation with high potassium. At stimulated synapses, the authors observed different membrane bending events, which the authors categorized and further tested, if there are other morphological differences like tethering/connecting synaptic vesicles, vesicle abundance. Based on the literature, they propose "early" and "late" exocytosis events without having any temporal resolution to distinguish between these two categories:
- The authors should add to the discussion, if and to what extent both exocytosis stages are expected under their stimulation

duration.
-Further, they should clearly point out the mentioned limitation: They are not stimulating with temporal precision (is mentioned) in such a way that early/late stages can be sorted according to any timepoints. The sorting only bases on morphological features at the active zone membrane. They need to claim that an uncertainty remains, if the observed and annotated structures are really exocytosis events and if this corresponds to late or early events. The annotation "late" and "early" represent a working hypothesis, which can be supported by the literature and other morphological differences as the connections/tethering/abundance of vesicles.
-I wondered, why is the membrane bending in Figure 3H was found with such a high frequency? Please discuss.
-Table EV1 should not claim "timepoint in ms", there is no millisecond-based annotation, maybe the proposed stages of the vesicles could be stated?
-Finally, one response in the author point-to-point raised a concern. The authors claimed that they used completely non-sprayed grids as controls, which means, as far as I understood, that these grids underwent a different experimental treatment than the sprayed ones. Could potential differences result from these different treatments? Please comment on that issue.

Dear Dr. Zuber,

Thank you for the submission of your revised manuscript. We have now received the enclosed report from referee 1 who was asked to assess it. Referee 1 still has a few more suggestions that I would like you to address and incorporate before we can proceed with the official acceptance of your manuscript. Please co-submit a point-by-point response to these last comments.

A few editorial requests will also need to be addressed:

- In the author checklist the questions on statistics should be answered. You can also just enter a note in the checklist on where in the ms this information can be found.

- Please add up to 5 keywords to the ms file.

- The REFERENCE FORMAT needs to be corrected to the named EMBO reports (Harvard) style.

- Funding information should only be included in the Acknowledgement section

- For figures Fig 2A, EV2A+B, EV3C-F, EV4A+B callouts are missing, please add.

- Figs 3,5,6 panel callouts are not alphabetically ordered, please correct.

- The movies and their legends should be ZIPd together and uploaded as one file per movie. The movie legends need to be removed from the manuscript file.

- The EV tables should be uploaded individually using the file type Expanded View (Expanded View Table 1, etc).

- The figure labeling needs correcting, i.e. Fig 1A1 should be Figure 1Ai.

- I attach to this email a related ms file with comments by our data editors. Please address all comments in the final ms.

EMBO press papers are accompanied online by A) a short (1-2 sentences) summary of the findings and their significance, B) 2-3 bullet points highlighting key results and C) a synopsis image that is exactly 550 pixels wide and 200-600 pixels high (the height is variable). You can either show a model or key data in the synopsis image. Please note that text needs to be readable at the final size. Please send us this information along with the revised manuscript.

Regarding the abstract, please double-check that it contains no overstatements. It currently says "time-resolved" and "early" and "late" fusion. If I understand the referees correctly, this must be corrected.

I look forward to seeing a final version of your manuscript as soon as possible. Please use this link to submit your revision: https://embor.msubmit.net/cgi-bin/main.plex

Best regards,
Esther

Esther Schnapp, PhD
Senior Editor
EMBO reports

Dear Dr Schnapp,
Thank you for your decision and forwarding referee #1's comments. We have addressed all the editorial requests above. Note that we have removed "time-resolved" from the abstract but we have left the terms early and late because they are not an overstatement. We just have added in the discussion an additional comment about how we defined early and late states in response to Referee #1 comment (see below).

Referee #1:

Radecke et al. significantly improved the quality of the manuscript. I highly appreciate that also new tomograms were acquired and included in the analysis.
Even though the exocytosis events remain rare, which might simply have biological reasons (could be discussed by the authors), the authors now better worked out the basis of sorting stimulated synapses in "late" and "early" staged exocytosis events.

We are grateful to the referee for their positive comments. Changes in the manuscript in response to the referee are highlighted in yellow.

However, some points remain to be clarified before publication:
In the end, the authors have a temporal range of stimulation with high potassium. At stimulated synapses, the authors observed different membrane bending events, which the authors categorized and further tested, if there are other morphological differences like tethering/connecting synaptic vesicles, vesicle abundance. Based on the literature, they propose "early" and "late" exocytosis events without having any temporal resolution to distinguish between these two categories:
- The authors should add to the discussion, if and to what extent both exocytosis stages are expected under their stimulation duration.

The complete collapse of SVs occurs somewhere between 20 and 50 ms after stimulation (Heuser and Reese 10.1083/jcb.88.3.564). Therefore, we could observe early and late stages of exocytosis on the same spray-mixed and plunge-frozen synaptosome grid.
We have included this in the discussion.

-Further, they should clearly point out the mentioned limitation: They are not stimulating with temporal precision (is mentioned) in such a way that early/late stages can be sorted according to any timepoints. The sorting only bases on morphological features at the active zone membrane. They need to claim that an uncertainty remains, if the observed and annotated structures are really exocytosis events and if this corresponds to late or early events. The annotation "late" and "early" represent a working hypothesis, which can be supported by the literature and other morphological differences as the connections/tethering/abundance of vesicles.

We have added a sentence in the discussion to make the reader aware of this fact.

-I wondered, why is the membrane bending in Figure 3H was found with such a high frequency? Please discuss.

A possible reason could be that a high KCl droplet landed on or very close to a high proportion of the imaged sprayed synaptosomes, leading to a long delay between stimulation and freezing. Yet we do not feel confident enough about the explanation to put it in the manuscript.

-Table EV1 should not claim "timepoint in ms", there is no millisecond-based annotation, maybe the proposed stages of the vesicles could be stated?

We thank the referee for spotting this inconsistency. We have modified the column title and the entries for the control tomograms (0 → "not stimulated")

-Finally, one response in the author point-to-point raised a concern. The authors claimed that they used completely non-sprayed grids as controls, which means, as far as I understood, that these grids underwent a different experimental treatment than the sprayed ones. Could potential differences result from these different treatments? Please comment on that issue.

We have added a few sentences about this concern in the discussion. It is unlikely that the differences are due to the mere fact of spraying. Plunge-freezing is done in a water-saturated environment to prevent sample drying and therefore water droplets are also likely to form on the control grids as well.

Benoît Zuber
University of Bern
Institute of Anatomy
Baltzerstrasse 2
Bern, BE 3012
Switzerland

Dear Dr. Zuber,

I am very pleased to accept your manuscript for publication in the next available issue of EMBO reports. Thank you for your contribution to our journal.

At the end of this email I include important information about how to proceed. Please ensure that you take the time to read the information and complete and return the necessary forms to allow us to publish your manuscript as quickly as possible.

As part of the EMBO publication's Transparent Editorial Process, EMBO reports publishes online a Review Process File to accompany accepted manuscripts. As you are aware, this File will be published in conjunction with your paper and will include the referee reports, your point-by-point response and all pertinent correspondence relating to the manuscript.

If you do NOT want this File to be published, please inform the editorial office within 2 days, if you have not done so already, otherwise the File will be published by default [contact: emboreports@embo.org]. If you do opt out, the Review Process File link will point to the following statement: "No Review Process File is available with this article, as the authors have chosen not to make the review process public in this case."

Thank you again for your contribution to EMBO reports and congratulations on a successful publication. Please consider us again in the future for your most exciting work.

Yours sincerely,

Esther Schnapp, PhD
Senior Editor
EMBO reports

****

THINGS TO DO NOW:

Please note that you will be contacted by Wiley Author Services to complete licensing and payment information. The required 'Page Charges Authorization Form' is available here: https://www.embopress.org/pb-assets/embo-site/er_apc.pdf - please download and complete the form and return to embopressproduction@wiley.com

EMBO Press participates in many Publish and Read agreements that allow authors to publish Open Access with reduced/no publication charges. Check your eligibility: https://authorservices.wiley.com/author-resources/Journal-Authors/open-access/affiliation-policies-payments/index.html

You will receive proofs by e-mail approximately 2-3 weeks after all relevant files have been sent to our Production Office; you should return your corrections within 2 days of receiving the proofs.

Please inform us if there is likely to be any difficulty in reaching you at the above address at that time. Failure to meet our deadlines may result in a delay of publication, or publication without your corrections.

All further communications concerning your paper should quote reference number EMBOR-2022-55719V3 and be addressed to emboreports@wiley.com.

Should you be planning a Press Release on your article, please get in contact with emboreports@wiley.com as early as possible, in order to coordinate publication and release dates.

EMBO Press Author Checklist

Corresponding Author Name: Benoît Zuber	
Journal Submitted to: EMBO Reports	
Manuscript Number: EMBOR-2022-55719V2	

USEFUL LINKS FOR COMPLETING THIS FORM
The EMBO Journal - Author Guidelines
EMBO Reports - Author Guidelines
Molecular Systems Biology - Author Guidelines
EMBO Molecular Medicine - Author Guidelines

Reporting Checklist for Life Science Articles (updated January 2022)
This checklist is adapted from Materials Design Analysis Reporting (MDAR) Checklist for Authors. MDAR establishes a minimum set of requirements in transparent reporting in the life sciences (see Statement of Task: 10.31222/osf.io/9sm4x). Please follow the journal's guidelines in preparing your manuscript.
Please note that a copy of this checklist will be published alongside your article.

Abridged guidelines for figures
1. Data
The data shown in figures should satisfy the following conditions:
- the data were obtained and processed according to the field's best practice and are presented to reflect the results of the experiments in an accurate and unbiased manner.
- ideally, figure panels should include only measurements that are directly comparable to each other and obtained with the same assay.
- plots include clearly labeled error bars for independent experiments and sample sizes. Unless justified, error bars should not be shown for technical replicates.
- if n<5, the individual data points from each experiment should be plotted. Any statistical test employed should be justified.
- Source Data should be included to report the data underlying figures according to the guidelines set out in the authorship guidelines on Data Presentation.

2. Captions
Each figure caption should contain the following information, for each panel where they are relevant:
- a specification of the experimental system investigated (eg cell line, species name).
- the assay(s) and method(s) used to carry out the reported observations and measurements.
- an explicit mention of the biological and chemical entity(ies) that are being measured.
- an explicit mention of the biological and chemical entity(ies) that are altered/varied/perturbed in a controlled manner.
- the exact sample size (n) for each experimental group/condition, given as a number, not a range;
- a description of the sample collection allowing the reader to understand whether the samples represent technical or biological replicates (including how many animals, litters, cultures, etc.).
- a statement of how many times the experiment shown was independently replicated in the laboratory.
- definitions of statistical methods and measures:
  - common tests, such as t-test (please specify whether paired vs. unpaired), simple χ2 tests, Wilcoxon and Mann-Whitney tests, can be unambiguously identified by name only, but more complex techniques should be described in the methods section;
  - are tests one-sided or two-sided?
  - are there adjustments for multiple comparisons?
  - exact statistical test results, e.g., P values = x but not P values < x;
  - definition of 'center values' as median or average;
  - definition of error bars as s.d. or s.e.m.

Please complete ALL of the questions below.
Select "Not Applicable" only when the requested information is not relevant for your study.

Materials

**Newly Created Materials**	**Information included in the manuscript?**	**In which section is the information available?** (Reagents and Tools Table, Materials and Methods, Figures, Data Availability Section)
New materials and reagents need to be available; do any restrictions apply?	Not Applicable	

**Antibodies**	**Information included in the manuscript?**	**In which section is the information available?** (Reagents and Tools Table, Materials and Methods, Figures, Data Availability Section)
For **antibodies** provide the following information:<br>- Commercial antibodies: RRID (if possible) or supplier name, catalogue number and or/clone number<br>- Non-commercial: RRID or citation	Not Applicable	

**DNA and RNA sequences**	**Information included in the manuscript?**	**In which section is the information available?** (Reagents and Tools Table, Materials and Methods, Figures, Data Availability Section)
**Short novel DNA or RNA including primers, probes**: provide the sequences.	Not Applicable	

**Cell materials**	**Information included in the manuscript?**	**In which section is the information available?** (Reagents and Tools Table, Materials and Methods, Figures, Data Availability Section)
**Cell lines:** Provide species information, strain. Provide accession number in repository **OR** supplier name, catalog number, clone number, and/**OR** RRID.	Not Applicable	
**Primary cultures:** Provide species, strain, sex of origin, genetic modification status.	Yes	Materials and Methods
Report if the cell lines were recently **authenticated** (e.g., by STR profiling) and tested for mycoplasma contamination.	Not Applicable	

**Experimental animals**	**Information included in the manuscript?**	**In which section is the information available?** (Reagents and Tools Table, Materials and Methods, Figures, Data Availability Section)
**Laboratory animals or Model organisms:** Provide species, strain, sex, age, genetic modification status. Provide accession number in repository **OR** supplier name, catalog number, clone number, **OR** RRID.	Yes	Materials and methods
**Animal observed in or captured from the field:** Provide species, sex, and age where possible.	Not Applicable	
Please detail **housing and husbandry conditions**.	Yes	Materials and methods

**Plants and microbes**	**Information included in the manuscript?**	**In which section is the information available?** (Reagents and Tools Table, Materials and Methods, Figures, Data Availability Section)
**Plants:** provide species and strain, ecotype and cultivar where relevant, unique accession number if available, and source (including location for collected wild specimens).	Not Applicable	
**Microbes:** provide species and strain, unique accession number if available, and source.	Not Applicable	

**Human research participants**	**Information included in the manuscript?**	**In which section is the information available?** (Reagents and Tools Table, Materials and Methods, Figures, Data Availability Section)
If collected and within the bounds of privacy constraints report on age, sex and gender or ethnicity for all study participants.	Not Applicable	

**Core facilities**	**Information included in the manuscript?**	**In which section is the information available?** (Reagents and Tools Table, Materials and Methods, Figures, Data Availability Section)
If your work benefited from core facilities, was their service mentioned in the acknowledgments section?	Yes	Acknowledgment

Design

Study protocol	Information included in the manuscript?	In which section is the information available? (Reagents and Tools Table, Materials and Methods, Figures, Data Availability Section)
If study protocol has been **pre-registered, provide DOI in the manuscript**. For clinical trials, provide the trial registration number **OR** cite DOI.	Not Applicable	
Report the **clinical trial registration number** (at ClinicalTrials.gov or equivalent), where applicable.	Not Applicable	

Laboratory protocol	Information included in the manuscript?	In which section is the information available? (Reagents and Tools Table, Materials and Methods, Figures, Data Availability Section)
Provide DOI OR other citation details if **external detailed step-by-step protocols** are available.	Not Applicable	

Experimental study design and statistics	Information included in the manuscript?	In which section is the information available? (Reagents and Tools Table, Materials and Methods, Figures, Data Availability Section)
Include a statement about **sample size** estimate even if no statistical methods were used.	Yes	figures
Were any steps taken to minimize the effects of subjective bias when allocating animals/samples to treatment (e.g. **randomization procedure**)? If yes, have they been described?	Yes	Materials and Methods.
Include a statement about **blinding** even if no blinding was done.	Yes	Materials and Methods.
Describe **inclusion/exclusion criteria** if samples or animals were excluded from the analysis. Were the criteria pre-established?<br><br>If sample or data points were omitted from analysis, report if this was due to attrition or intentional exclusion and provide justification.	Yes	Materials and Methods.
For every figure, are **statistical tests** justified as appropriate? Do the data meet the assumptions of the tests (e.g., normal distribution)? Describe any methods used to assess it. Is there an estimate of variation within each group of data? Is the variance similar between the groups that are being statistically compared?	Yes	Materials and Methods.

Sample definition and in-laboratory replication	Information included in the manuscript?	In which section is the information available? (Reagents and Tools Table, Materials and Methods, Figures, Data Availability Section)
In the figure legends: state number of times the experiment was **replicated** in laboratory.	Not Applicable	
In the figure legends: define whether data describe **technical or biological replicates**.	Not Applicable	

Ethics

Ethics	Information included in the manuscript?	In which section is the information available? (Reagents and Tools Table, Materials and Methods, Figures, Data Availability Section)
Studies involving **human participants**: State details of **authority granting ethics approval** (IRB or equivalent committee(s), provide reference number for approval.	Not Applicable	
Studies involving **human participants**: Include a statement confirming that **informed consent** was obtained from all subjects and that the experiments conformed to the principles set out in the WMA Declaration of Helsinki and the Department of Health and Human Services Belmont Report.	Not Applicable	
Studies involving **human participants:** For publication of **patient photos**, include a statement confirming that consent to publish was obtained.	Not Applicable	
Studies involving experimental **animals**: State details of **authority granting ethics approval** (IRB or equivalent committee(s), provide reference number for approval. Include a statement of compliance with ethical regulations.	Yes	Material and Methods
Studies involving **specimen and field samples:** State if relevant **permits** obtained, provide details of authority approving study; if none were required, explain why.	Not Applicable	

Dual Use Research of Concern (DURC)	Information included in the manuscript?	In which section is the information available? (Reagents and Tools Table, Materials and Methods, Figures, Data Availability Section)
Could your study fall under dual use research restrictions? Please check biosecurity documents and list of **select agents and toxins** (CDC): https://www.selectagents.gov/sat/list.htm	Not Applicable	
If you used a select agent, is the security level of the lab appropriate and reported in the manuscript?	Not Applicable	
If a study is subject to dual use research of concern regulations, is the name of the **authority granting approval and reference number** for the regulatory approval provided in the manuscript?	Not Applicable	

Reporting

The MDAR framework recommends adoption of discipline-specific guidelines, established and endorsed through community initiatives. Journals have their own policy about requiring specific guidelines and recommendations to complement MDAR.

Adherence to community standards	Information included in the manuscript?	In which section is the information available? (Reagents and Tools Table, Materials and Methods, Figures, Data Availability Section)
State if relevant guidelines or checklists (e.g., **ICMJE, MIBBI, ARRIVE, PRISMA**) have been followed or provided.	Not Applicable	
For **tumor marker prognostic studies,** we recommend that you follow the **REMARK** reporting guidelines (see link list at top right). See author guidelines, under 'Reporting Guidelines'. Please confirm you have followed these guidelines.	Not Applicable	
For **phase II and III randomized controlled trials**, please refer to the **CONSORT** flow diagram (see link list at top right) and submit the CONSORT checklist (see link list at top right) with your submission. See author guidelines, under 'Reporting Guidelines'. Please confirm you have submitted this list.	Not Applicable	

Data Availability

Data availability	Information included in the manuscript?	In which section is the information available? (Reagents and Tools Table, Materials and Methods, Figures, Data Availability Section)
Have **primary datasets** been deposited according to the journal's guidelines (see 'Data Deposition' section) and the respective accession numbers provided in the Data Availability Section?	Not Applicable	
Were **human clinical and genomic datasets** deposited in a public access-controlled repository in accordance to ethical obligations to the patients and to the applicable consent agreement?	Not Applicable	
Are **computational models** that are central and integral to a study available without restrictions in a machine-readable form? Were the relevant accession numbers or links provided?	Not Applicable	
If publicly available data were reused, provide the respective **data citations in the reference list**.	Not Applicable	
